# Glycogen metabolism regulates macrophage-mediated acute inflammatory responses

**DOI:** 10.1038/s41467-020-15636-8

**Published:** 2020-04-14

**Authors:** Jingwei Ma, Keke Wei, Junwei Liu, Ke Tang, Huafeng Zhang, Liyan Zhu, Jie Chen, Fei Li, Pingwei Xu, Jie Chen, Jincheng Liu, Haiqing Fang, Liang Tang, Dianheng Wang, Liping Zeng, Weiwei Sun, Jing Xie, Yuying Liu, Bo Huang

**Affiliations:** 1 Department of Immunology, Tongji Medical College, Huazhong University of Science & Technology, Wuhan, 430030 China; 2 Department of Biochemistry & Molecular Biology, Tongji Medical College, Huazhong University of Science & Technology, Wuhan, 430030 China; 3 Cardiovascular Surgery, Union Hospital, Huazhong University of Science & Technology, Wuhan, 430071 China; 4 Department of Immunology & National Key Laboratory of Medical Molecular Biology, Institute of Basic Medical Sciences, Chinese Academy of Medical Sciences (CAMS) & Peking Union Medical College, Beijing, 100005 China; 5 Clinical Immunology Center, CAMS, Beijing, 100005 China

**Keywords:** Glycobiology, Monocytes and macrophages, Infectious diseases

## Abstract

Our current understanding of how sugar metabolism affects inflammatory pathways in macrophages is incomplete. Here, we show that glycogen metabolism is an important event that controls macrophage-mediated inflammatory responses. IFN-γ/LPS treatment stimulates macrophages to synthesize glycogen, which is then channeled through glycogenolysis to generate G6P and further through the pentose phosphate pathway to yield abundant NADPH, ensuring high levels of reduced glutathione for inflammatory macrophage survival. Meanwhile, glycogen metabolism also increases UDPG levels and the receptor P2Y_14_ in macrophages. The UDPG/P2Y_14_ signaling pathway not only upregulates the expression of STAT1 via activating RARβ but also promotes STAT1 phosphorylation by downregulating phosphatase TC45. Blockade of this glycogen metabolic pathway disrupts acute inflammatory responses in multiple mouse models. Glycogen metabolism also regulates inflammatory responses in patients with sepsis. These findings show that glycogen metabolism in macrophages is an important regulator and indicate strategies that might be used to treat acute inflammatory diseases.

## Abstract (web summary)

Glycogen can be metabolized via glycogenolysis and the pentose phosphate pathway as well as into the production of UDP glucose, which when secreted can bind the P2Y_14_ receptor. Here the authors show how these glycogen metabolism pathways contribute to proinflammatory macrophage activation and susceptibility to sepsis.

## Introduction

Macrophages can be polarized to an inflammatory phenotype by interferon-γ (IFN-γ) and lipopolysaccharide (LPS) or to an anti-inflammatory phenotype by interleukin-4 (IL-4) or other factors. Although inflammatory macrophages play a crucial role in phagocytosis and eliminating bacteria, uncontrolled activation of inflammatory macrophages may trigger systemic inflammatory response syndrome (SIRS) or even sepsis^1–3^. Therefore, precise regulation of inflammatory macrophages is of paramount importance in guaranteeing microbial clearance, injury limitation, and avoidance of serious side effects^4,5^. It is known that inflammatory phenotype of macrophages is profoundly regulated by glucose metabolism^6–8^, however this metabolic regulation remains incompletely understood. In addition to glycolysis as the mark for inflammatory metabolic phenotype^9,10^, macrophages have recently been shown to utilize glucose metabolic reprograms such as pentose phosphate pathway (PPP) and mitochondrial succinate oxidation to drive their inflammatory phenotype^11–13^. But how the glucose metabolic cues trigger inflammatory gene expression in macrophages remains unclear. In addition, whether and how other glucose-related metabolic pathway(s) regulates inflammatory macrophages has not been well studied.

Glycogen is an important carbohydrate fuel reserve and glycogen metabolism has been previously reported in myeloid cells of the immune system including dendritic cells and macrophages^14–16^. Our recent studies additionally found that CD8^+^ memory T cells actively mobilize glycogen metabolism to generate glucose-6-phosphate (G6P) via glycogenolysis and channel the G6P to the PPP^17^. In this metabolic model, the glycogen is not primarily used to store energy, but to provide antioxidant defense through the generation of NADPH and subsequently reduced glutathione. These findings imply that glycogen may exert important biological effects through metabolic pathways beyond its role as a reservoir of glucose. In support of this notion, UDP-glucose (UDPG), a glycogen metabolic intermediate, has been shown to act as a ligand for purigenic receptor P2Y_14_, thus directly triggering signal transduction^18–20^.

In the present study, we provide evidence that glycogen metabolism has a central function in controlling inflammatory pathways of macrophages by two related mechanisms: firstly, macrophages use glycolysis-derived G6P to synthesize glycogen, and subsequent glycogenolysis regenerates G6P which is channeled through the PPP to produce large amounts of NADPH required for inflammatory macrophage survival; secondly, glycogenesis-derived UDPG activates the P2Y_14_ receptor, whose signaling transduction regulates the inflammatory phenotypes of macrophages in an autocrine fashion.

## Results

### Glycogen is synthesized in inflammatory macrophages

**Fig. 1:**
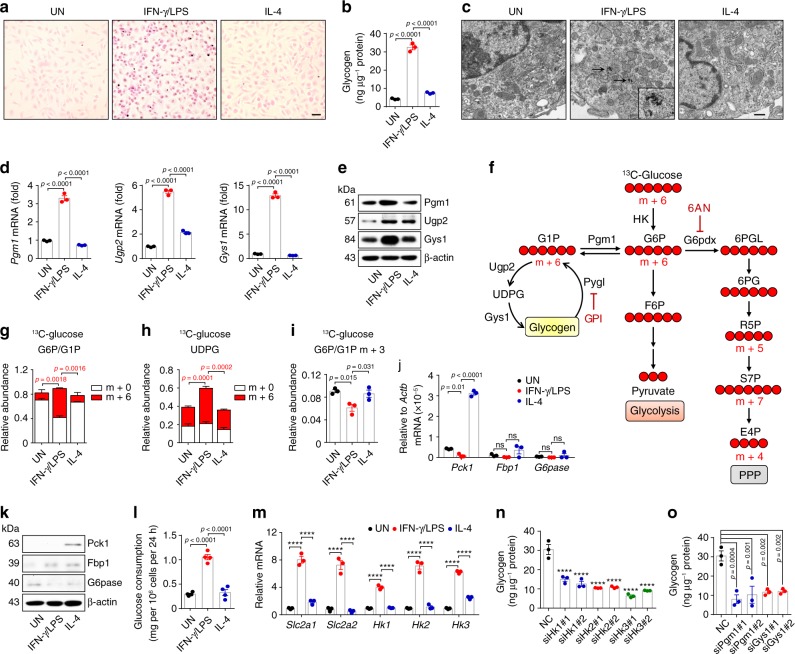
Glycogen is synthesized in inflammatory macrophages. **a**–**c** Intracellular glycogen levels in untreated, IFN-γ/LPS (20 ng mL^−1^ IFN-γ plus 100 ng mL^−1^ LPS) or IL-4 (10 ng mL^−1^) treated BMDMs were detected and observed by PAS staining (scale bar, 20 μm) (**a**), colorimetric assay (**b**) and TEM (scale bar, 0.5 μm) (**c**). The arrows point to intracellular glycogen deposits. **d**, **e** Pgm1, Ugp2, and Gys1 expression in untreated, IFN-γ/LPS or IL-4 treated BMDMs were determined by real-time PCR (**d**) and western blot (**e**). **f** Overview of three glucose metabolic pathways: glycogen metabolism (left), glycolysis (middle) and PPP (right) and the inhibitors (GPI/6AN) are shown. **g**–**i** BMDMs differentiated in normal ^12^C-glucose were stimulated with IFN-γ/LPS or IL-4 for 6 h and switched to ^13^C-glucose for 6 h, LC-MS/MS was performed for m + 6-labeled G6P/G1P (**g**), m + 6-labeled UDPG (**h**) and m + 3-labeled G6P/G1P (**i**). **j**, **k** Pck1, Fbp1, and G6pase expression in untreated, IFN-γ/LPS or IL-4 treated BMDMs were determined by real-time PCR (**j**) and western blot (**k**). **l** Consumption of glucose in untreated, IFN-γ/LPS or IL-4 treated BMDMs were measured by enzymatic methods. **m** Relative mRNA expression of *Slc2a1/2* and *Hk1/2/3* in untreated, IFN-γ/LPS or IL-4 treated BMDMs were determined by real-time PCR. **n**, **o** *Hk1/2/3*, *Pgm1* or *Gys1* siRNA transfected BMDMs were stimulated with IFN-γ/LPS for 36 h. Intracellular glycogen levels were detected by colorimetric assay. Unless otherwise specified, *n* = 3 biologically independent experiments were performed. Data are presented as mean ± SEM. *P* values were calculated using one-way ANOVA, ****p < 0.0001.

To test the possible role of glycogen in inflammatory macrophages, we first determined whether glycogen was synthesized in the cells. We cultured murine bone marrow-derived untreated, IFN-γ/LPS-treated inflammatory and IL-4 treated anti-inflammatory macrophages, respectively. We found that glycogen was strikingly increased in the inflammatory macrophages, as evidenced by PAS staining and colorimetric assay (Fig. 1a, b), which was further confirmed by transmission electron microscope (TEM) (Fig. 1c). In line with these results, enzymes involved in glycogen biosynthesis, including phosphoglucomutase 1 (Pgm1), UDP-glucose pyrophosphorylase 2 (Ugp2, the catalytic enzyme converting G1P to UDPG) and glycogen synthase 1 (Gys1), were upregulated in inflammatory macrophages and only Ugp2 was slightly increased a lesser extent by IL-4 (Fig. 1d, e). To further confirm the above result, we added [U^6^]-^13^C-glucose to the cultured medium of macrophages (Fig. 1f). The ^13^C carbon tracing showed a striking increase of m + 6 G6P/G1P, concomitant with higher levels of m + 6 UDPG in the inflammatory macrophages, compared to controls (Fig. 1g, h), indicating a potential presence of an active glycogen synthesis in the inflammatory macrophages. To validate the above murine data in human macrophages, we additionally repeated the experiments in human monocytic THP-1 cells. Similarly, IFN-γ/LPS-treated THP-1 cells displayed much higher glycogen levels, concomitant with the upregulation of enzymes involved in glycogen synthesis (Supplementary Fig. 1a–c). Moreover, we cultured bone marrow cells in the ^13^C-glucose culture medium and induced them to differentiate to macrophages for 5 days. The cells were then stimulated with IFN-γ/LPS for 24 h, followed by the treatment with hydrochloric acid to degrade polymer glycogen into monomer glucose^21^. The released ^13^C-labeled glucose was determined by LC-MS/MS, which showed a result of 70% ^13^C-labeled glucose (Supplementary Fig. 1d). These results suggest that inflammatory macrophages use glycogen as a metabolic way.

**Fig. 2:**
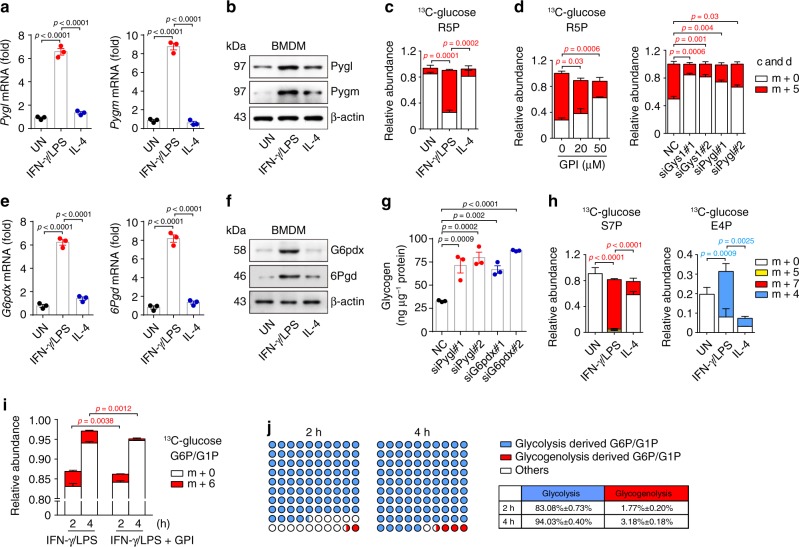
Glycogenolysis-derived G6P is channeled to the PPP. **a**, **b** Pygl and Pygm expression in untreated, IFN-γ/LPS or IL-4 treated BMDMs were determined by real-time PCR (**a**) and western blot (**b**). **c** BMDMs differentiated in normal ^12^C-glucose were stimulated with IFN-γ/LPS or IL-4 for 6 h and switched to ^13^C-glucose for 6 h, LC-MS/MS was performed for m + 5-labeled R5P. **d** BMDMs were pretreated with GPI for 30 min or pretransfected with siRNA (*Gys1* or *Pygl*) for 24 h prior to stimulation with IFN-γ/LPS for 6 h and switched to ^13^C-glucose for 6 h, LC-MS/MS was performed for m + 5-labeled R5P. **e**, **f** G6pdx and 6Pgd expression in untreated, IFN-γ/LPS or IL-4 treated BMDMs were determined by real-time PCR (**e**) and western blot (**f**). **g** *Pygl* or *G6pdx* siRNA transfected BMDMs were stimulated with IFN-γ/LPS for 36 h. Intracellular glycogen levels were detected by colorimetric assay. **h** BMDMs differentiated in normal ^12^C-glucose were stimulated with IFN-γ/LPS or IL-4 for 6 h and switched to ^13^C-glucose for 6 h, LC-MS/MS was performed for m + 7-labeled S7P, m + 5-labeled S7P and m + 4-labeled E4P. **i**, **j** BMDMs cultured and differentiated in ^13^C-glucose were pretreated with GPI for 30 min prior to stimulation with IFN-γ/LPS for 6 h and switched to ^12^C-glucose for 2 or 4 h, ^13^C-labeled G6P/G1P were detected by LC-MS/MS (**i**). The ratio of G6P/G1P from glycogenolysis or glycolysis was calculated. The glycogenolysis-derived G6P using the format of [m + 6 G6P (IFN-γ/LPS)–m + 6 G6P (IFN-γ/LPS + GPI)]/Total G6P (IFN-γ/LPS) and the glycolysis-derived G6P by the format of [m + 0 G6P (IFN-γ/LPS)] / Total G6P (IFN-γ/LPS) (**j**). Unless otherwise specified, *n* = 3 biologically independent experiments were performed. Data are presented as mean ± SEM. *P* values were calculated using one-way ANOVA.

Glycogen synthesis is initiated from the transition of G6P to G1P. Several pathways can be the source of G6P, including gluconeogenesis, glycogenolysis, and glycolysis^22,23^. Previously, we have shown that CD8^+^ memory T cells use gluconeogenesis to generate G6P^17^. However, this seemed not to be the case in macrophages, because (1) the ^13^C-glucose tracing did not show m + 3 G6P/G1P to reflect gluconeogenesis (Fig. 1i); (2) ^13^C-pyruvate or ^13^C-acetate tracing did not show m + 2 G6P/G1P (Supplementary Fig. 1e, f); and (3) key gluconeogenic enzymes phosphoenolpyruvate carboxykinase 1 (Pck1) and fructose-1,6-bisphosphatase (Fbp1) were not upregulated; instead they were slightly downregulated (Fig. 1j, k). In addition, to avoid a futile metabolism, G6P should not be derived from glycogenolysis for glycogen biosynthesis. Thus, for inflammatory macrophages, G6P was likely derived from the glycolysis. Indeed, the macrophages consumed more glucose, upregulated glucose transporter *Slc2a1/2* and enzyme hexokinase (*Hk1/2/3*), leading to the increased production of G6P (Fig. 1l, m). By contrast, knocking down *Hk1/2/3* to inhibit glycolysis-derived G6P reduced the glycogen levels in inflammatory macrophages (Fig. 1n and Supplementary Fig. 1g). Also, the knockdown of *Pgm1* or *Gys1* resulted in the decreased glycogen levels in inflammatory macrophages (Fig. 1o and Supplementary Fig. 1g). Together, these data suggest that inflammatory macrophages mobilize glycolysis-derived G6P to initiate glycogen synthesis.

### Glycogenolysis-derived G6P is channeled to the PPP

Synthesized glycogen is stored in the cytoplasm or enters glycogenolysis for degradation^24^. Notably, glycogen-degrading enzymes such as glycogen phosphorylase Pygl (liver) and Pygm (muscle) were found to be upregulated in IFN-γ/LPS-treated rather than untreated or IL-4-treated macrophages (Fig. 2a, b). Consistent results were also obtained from IFN-γ/LPS-treated human THP-1 cells (Supplementary Fig. 2a, b), implying that inflammatory macrophages have glycogenolytic activity, leading to G6P production. In addition, we roughly calculated the glycogen turnover rate, which was around 52% (Supplementary Fig. 2c). As a central metabolite, G6P can be channeled to different directions: becoming glucose via dephosphorylation; being oxidized to pyruvate along glycolysis or to ribose-5-phosphate (R5P) via PPP^22,23^. The ^13^C tracing showed that G6P could be channeled to m + 5 R5P (Fig. 2c), which was blocked by glycogen phosphorylase inhibitor (GPI), *Gys1* or *Pygl* siRNA (Fig. 2d), suggesting that glycogenolysis-derived G6P is channeled through the PPP. Consistently, two enzymes G6P dehydrogenase (G6pdx) and 6-phosphogluconate dehydrogenase (6Pgd) that mediate the oxidation of PPP were upregulated in inflammatory macrophages (Fig. 2e, f). Blocking PPP by *G6pdx* siRNA or G6pdx inhibitor 6-aminonicotinamide (6AN) or blocking glycogenolysis by *Pygl* siRNA or GPI led to accumulation of glycogen in inflammatory macrophages (Fig. 2g and Supplementary Fig. 2d, e). The PPP can be divided into oxidative and non-oxidative steps: G6P is first oxidized to an intermediate molecule ribulose 5-phosphate (Ru5P); for the non-oxidative step, Ru5P is either converted to R5P for nucleotide synthesis^25^, or converted to R5P and xylulose 5-phosphate (X5P), leading to the generation of intermediate products [sedoheptulose 7-phosphate (S7P) and erythrose 4-phosphate (E4P)] and end products [glyceraldehyde 3-phosphate (G3P) and fructose 6-phosphate (F6P)]^26^. In line with the carbon flow from G6P to R5P, the ^13^C tracing assay further showed that G6P could be channeled to m + 7 S7P and m + 4 E4P (Fig. 2h). Blocking glycogen synthesis by *Ugp2* or *Gys1* siRNA or blocking glycogenolysis by *Pygl* siRNA led to decreased S7P and E4P in inflammatory macrophages (Supplementary Fig. 2f), suggesting that glycogenolysis-derived G6P is channeled through the PPP in inflammatory macrophages. Here, we also clarified how much G6P was derived from glucose taken up by the macrophages versus how much G6P was generated from glycogenolysis. Bone marrow cells were cultured with [U6]-^13^C-glucose medium for 5 days in the presence of M-CSF, followed by 6-hour stimulation with IFN-γ/LPS or IFN-γ/LPS + GPI and the switch of the medium to ^13^C-glucose-free medium for the last 2- or 4 h. Cell lysates were then analyzed by LC-MS/MS. Based on such m + 6 G6P tracing, we calculated that 83.08% vs. 1.77% G6P at 2 h and 94.03% vs. 3.18% G6P at 4 h were generated by glycolysis vs. glycogenolysis (Fig. 2i, j). In addition, we found that blockade of glycogenolysis by GPI led to the increase of ^13^C-labeled glucose in glycogen from 70 to 84% and the decrease of m + 5 R5P from 95% to 84% (Supplementary Fig. 2g). This 14% increase was some consistent with 11% decrease, suggesting that glycogenolysis-derived G6P might flow to PPP.

### PPP regulates macrophage phenotype, function, and survival

**Fig. 3:**
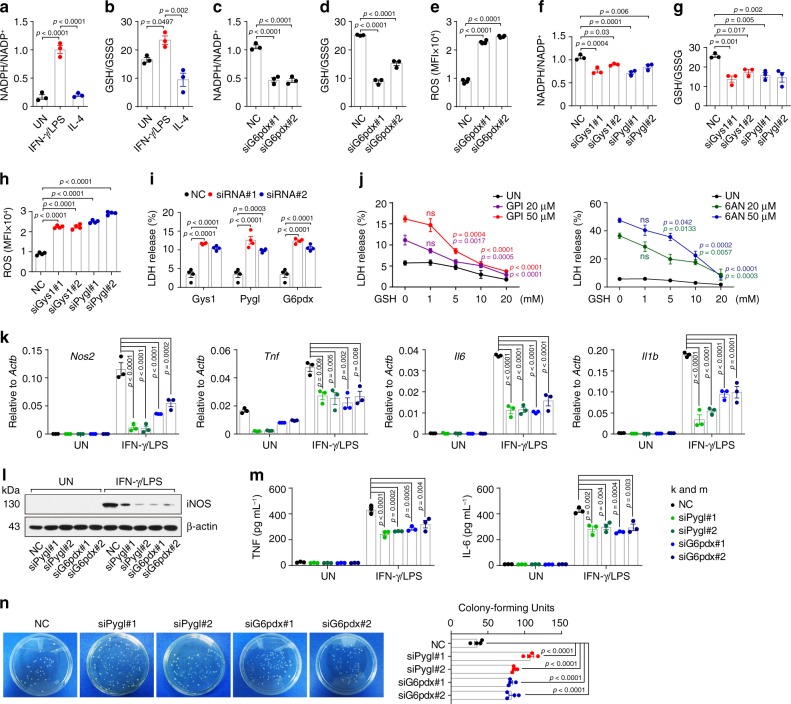
PPP regulates macrophage phenotype, function and survival. **a**, **b** NADPH/NADP^+^ (**a**) and GSH/GSSG (**b**) ratio in untreated, IFN-γ/LPS or IL-4 treated BMDMs were analyzed. **c**–**e** *G6pdx* siRNA transfected BMDMs were stimulated with IFN-γ/LPS for 24 h, NADPH/NADP^+^ (**c**), GSH/GSSG (**d**) and ROS (**e**) were analyzed. MFI, mean fluorescence intensity. **f**–**h** *Gys1* or *Pygl* siRNA transfected BMDMs were stimulated with IFN-γ/LPS for 24 h, NADPH/NADP^+^ (**f**), GSH/GSSG (**g**) and ROS (**h**) were analyzed. **i** *Gys1*, *Pygl* or *G6pdx* siRNA transfected BMDMs were stimulated with IFN-γ/LPS for 24 h, lactic dehydrogenase (LDH) release was analyzed. **j** BMDMs were pretreated with GPI or 6AN for 30 min prior to stimulation with IFN-γ/LPS ± GSH (0, 1, 5, 10, 20 mM) for 36 h, and LDH release was analyzed. **k**–**m** *Pygl* or *G6pdx* siRNA transfected BMDMs were stimulated with or without IFN-γ/LPS for 24 or 36 h, *Nos2*, *Tnf*, *Il6,* and *Il1b* expression was determined by real-time PCR (**k**), iNOS, TNF and IL-6 expression was determined by western blot (**l**) and ELISA (**m**). **n** *Pygl* or *G6pdx* siRNA transfected BMDMs were stimulated with IFN-γ/LPS for 24 h and incubated with E.coli at ratio 1:1 for 3 h, then the *E.coli* was collected, coated and cultured with ampicillin-resistant agarose solid medium at 37 °C for 36 h, followed by colony-forming units count. Data are presented as mean ± SEM of *n* = 3 biologically independent experiments (**a**–**d**, **f**, **g**, **j**, **k**, and **m**) or *n* = 4 biologically independent experiments (**e**, **h**, **i** and **n**). *P* values were calculated using one-way ANOVA.

One biological significance of the PPP lies in the generation of NADPH, which is crucial for the reduction of oxidized glutathione to GSH, thus maintaining the redox homeostasis of cells^27–29^. As expected, higher levels of NADPH were found in IFN-γ/LPS-treated macrophages (Fig. 3a), concomitant with the higher ratio of GSH/GSSG relative to that in control macrophages (Fig. 3b). Blocking the PPP by either *G6pdx* siRNA or 6AN effectively decreased NADPH levels and the GSH/GSSG ratio (Fig. 3c, d and Supplementary Fig. 3a, b), but increased ROS levels in the macrophages (Fig. 3e and Supplementary Fig. 3c). Consistently, blocking glycogen metabolism by *Gys1* or *Pygl* siRNA or GPI also led to decreased NADPH levels, GSH/GSSG ratio and increased ROS levels (Fig. 3f–h and Supplementary Fig. 3d, e). In line with increased ROS levels, lactate dehydrogenase (LDH) was strikingly present in the supernatants, reflecting a state of cell death (Fig. 3i). This release of LDH, however, could be inhibited by the supply of exogenous GSH (Fig. 3j). Notably, disruption of either PPP or glycogenolysis by siRNA or inhibitor resulted in the downregulation of the expression of iNOS, TNF, IL-6 and *Il1b* in IFN-γ/LPS-treated macrophages (Fig. 3k-m and Supplementary Fig. 3f, g), concomitant with decreased ability to clear bacteria (Fig. 3n). Together, these data suggest that the glycogen-PPP metabolic pathway regulates the phenotype, function and survival of inflammatory macrophages.

### UDPG regulates inflammatory macrophages via P2Y_14_ receptor

**Fig. 4:**
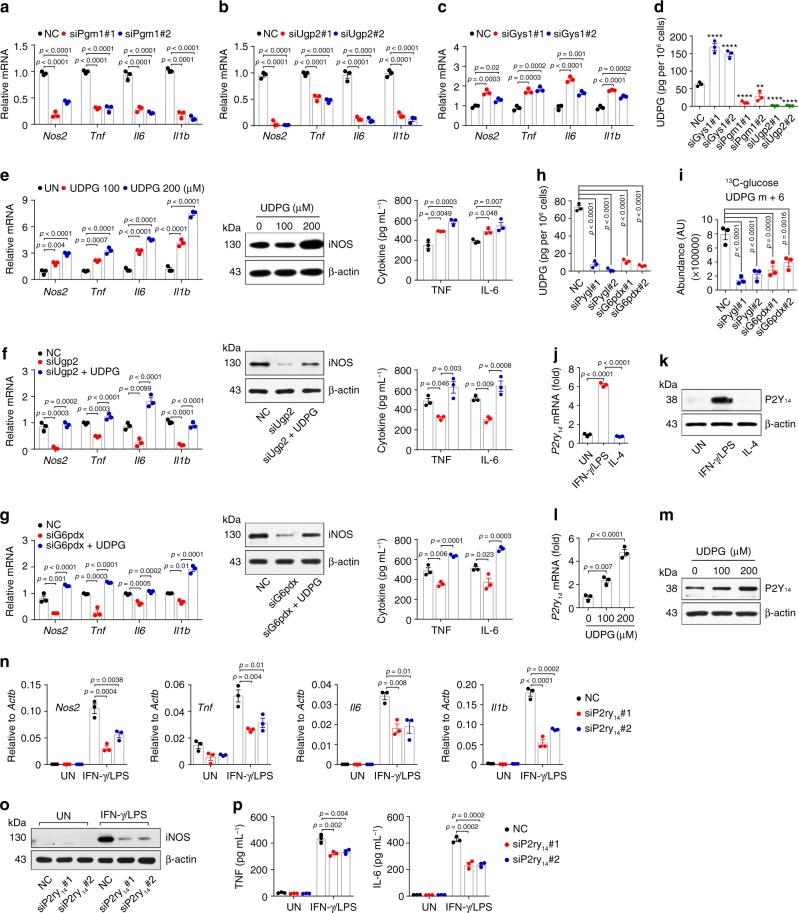
UDPG regulates inflammatory macrophages via P2Y_14_ receptor. **a**–**c** *Pgm1*, *Ugp2* or *Gys1* siRNA transfected BMDMs were stimulated with IFN-γ/LPS for 24 h, *Nos2*, *Tnf*, *Il6* and *Il1b* expression was determined by real-time PCR. **d** *Gys1*, *Pgm1* or *Ugp2* siRNA transfected BMDMs were stimulated with IFN-γ/LPS for 24 h, UDPG in supernatants was determined by ELISA. **e** IFN-γ/LPS-stimulated BMDMs were treated with UDPG (0, 100 or 200 μM) for 24 or 36 h, *Nos2*, *Tnf*, *Il6,* and *Il1b* expression was determined by real-time PCR (left), iNOS, TNF and IL-6 expression was determined by western blot (middle) and ELISA (right). **f**, **g** *Ugp2* or *G6pdx* siRNA transfected BMDMs were stimulated with IFN-γ/LPS ± UDPG (200 μM) for 24 or 36 h, *Nos2*, *Tnf*, *Il6,* and *Il1b* expression was determined by real-time PCR (left), iNOS, TNF and IL-6 expression was determined by western blot (middle) and ELISA (right). **h** *Pygl* or *G6pdx* siRNA transfected BMDMs were stimulated with IFN-γ/LPS for 24 h, UDPG in supernatants was determined by ELISA. **i** BMDMs were pretransfected with siRNA (*Pygl* or *G6pdx*) for 24 h prior to stimulation with IFN-γ/LPS for 6 h and switched to ^13^C-glucose for 6 h, lysed cells were analyzed by LC-MS/MS to determine m + 6-labeled UDPG. **j**, **k** P2Y_14_ receptor expression in untreated, IFN-γ/LPS- or IL-4-treated BMDMs was determined by real-time PCR (**j**) and western blot (**k**). **l**, **m** IFN-γ/LPS-stimulated BMDMs were treated with UDPG (0, 100 or 200 μM) for 24 or 36 h, P2Y_14_ expression was determined by real-time PCR (**l**) and western blot (**m**). **n**–**p** *P2ry*_*14*_ siRNA transfected BMDMs were stimulated with or without IFN-γ/LPS for 24 or 36 h, *Nos2*, *Tnf*, *Il6,* and *Il1b* expression was determined by real-time PCR (**n**), iNOS, TNF, and IL-6 expression was determined by western blot (**o**) and ELISA (**p**). Unless otherwise specified, *n* = 3 biologically independent experiments were performed. Data are presented as mean ± SEM. *P* values were calculated using one-way ANOVA. **p < 0.01, ****p < 0.0001.

Although the above results showed that the inhibition of glycogenolysis downregulated the expression of proinflammatory genes in IFN-γ/LPS-treated macrophages, blocking glycogen synthesis at different stages produced conflicting results. Blocking the conversion of G6P to G1P by *Pgm1* siRNA or G1P to UDPG by *Ugp2* siRNA resulted in the downregulation of the above-mentioned proinflammatory genes (Fig. 4a, b); unexpectedly, those genes, however, were upregulated by knocking down *Gys1*, the enzyme transferring the glucose group from UDPG to glycogen (Fig. 4c). This apparent paradox can likely be ascribed to the metabolite molecule UDPG in that UDPG levels were increased by *Gys1* siRNA but decreased by *Pgm1* or *Ugp2* siRNA (Fig. 4d). Moreover, the supplement of exogenous UDPG not only upregulated those inflammatory genes expression in inflammatory macrophages (Fig. 4e), but also rescued the downregulated genes in the *Ugp2* siRNA group (Fig. 4f and Supplementary Fig. 4a), suggesting that glycogenesis-derived UDPG regulates the inflammatory phenotype of macrophages. In addition, UDPG also rescued the downregulated inflammatory genes in the *G6pdx* siRNA group (Fig. 4g). Despite the increase of UDPG levels by the inhibition of glycogen synthesis, blocking glycogenolysis or PPP however resulted in decreased UDPG levels, as evidenced by the ELISA detection and the ^13^C carbon tracing (Fig. 4h, i and Supplementary Fig. 4b). Intriguingly, we found that the inhibition of glycogen synthesis led to upregulation expression of *Pgm1* and *Ugp2*, however the inhibition of glycogenolysis or PPP led to downregulation expression of *Pgm1* and *Ugp2* (Supplementary Fig. 4c, d), thus explaining the inconsistence. It is known that UDPG could be released from Golgi to the extracellular space through a secretory pathway^30,31^, where UDPG bound to the receptor P2Y_14_ for signal transduction^18–20,31,32^. Notably, the expression of P2Y_14_ receptor was upregulated in IFN-γ/LPS-treated macrophages (Fig. 4j, k), and further enhanced by exogenous UDPG addition (Fig. 4l, m). By contrast, the expression of *P2ry*_*14*_ was downregulated in *Pygl* or *Ugp2* siRNA-treated inflammatory macrophages (Supplementary Fig. 4e). Moreover, when we used siRNA to knock down P2Y_14_ receptor (Supplementary Fig. 4f), we found that those inflammatory genes were downregulated in IFN-γ/LPS-treated macrophages (Fig. 4n–p). A similar result was also obtained by using P2Y_14_ receptor antagonist PPTN (Supplementary Fig. 4g–i). In addition, the addition of UDPG did not upregulate the expression of P2Y_14_, STAT1 and pro-inflammatory cytokines in IL-4-stimulated macrophages (Supplementary Fig. 4j–l). Together, these results suggest that UDPG regulates inflammatory macrophage phenotype via the P2Y_14_ receptor.

### UDPG/P2Y_14_ regulates STAT1 expression and phosphorylation

**Fig. 5:**
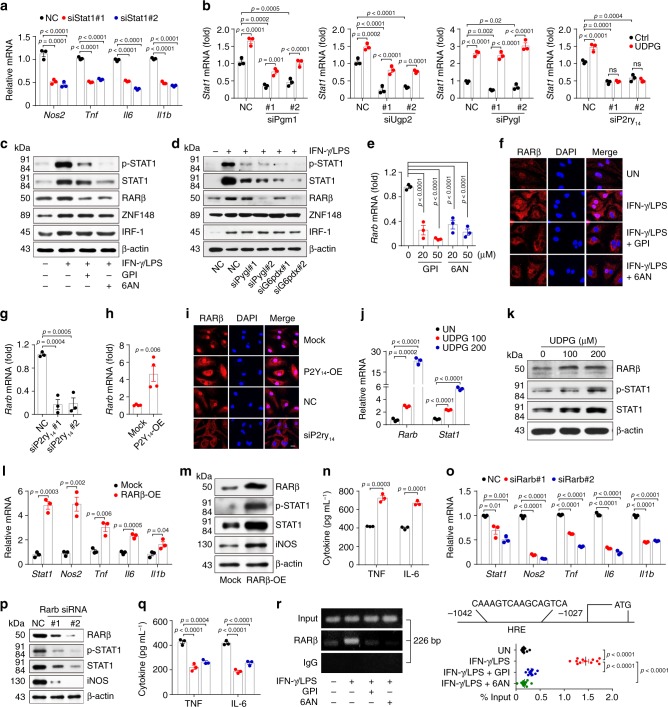
UDPG-P2Y_14_ pathway regulates STAT1 expression. **a** *Stat1* siRNA transfected BMDMs were stimulated with IFN-γ/LPS for 24 h, *Nos2*, *Tnf*, *Il6,* and *Il1b* expression was determined by real-time PCR. **b** *Pgm1*, *Ugp2*, *Pygl* or *P2ry*_*14*_ siRNA transfected BMDMs were stimulated with IFN-γ/LPS ± UDPG for 24 h, *Stat1* expression was determined by real-time PCR. **c**–**f** BMDMs were pretreated with inhibitor (GPI or 6AN) for 30 min or pre-transfected with siRNA (*Pygl* or *G6pdx*) for 24 h prior to stimulation with IFN-γ/LPS for 24 or 36 h, STAT1, ZNF-148, IRF-1, and RARβ expression was determined by western blot (**c**, **d**), RARβ expression and location were analyzed by real-time PCR (**e**) and confocal microscope, scale bar, 10 μm (**f**). **g**–**i** *P2ry*_*14*_ siRNA or P2Y_14_-overexpression vectors (P2Y_14_-OE) transfected BMDMs were stimulated with IFN-γ/LPS for 24 or 36 h, RARβ expression and location were determined by real-time PCR (**g**, **h**) and confocal microscope, scale bar, 10 μm (**i**). **j**, **k** IFN-γ/LPS stimulated BMDMs were treated with UDPG for 24 or 36 h, RARβ and STAT1 expression was determined by real-time PCR (**j**) and western blot (**k**). **l**–**q** RARβ-overexpression vectors (RARβ-OE) or *Rarb* siRNA transfected BMDMs were stimulated with IFN-γ/LPS for 24 or 36 h, *Stat1*, *Nos2*, *Tnf*, *Il6,* and *Il1b* expression was determined by real-time PCR (**l**, **o**), RARβ, STAT1, iNOS, TNF, and IL-6 expression was determined by western blot (**m**, **p**) and ELISA (**n**, **q**). **r** Schematic representation of the promoter region on the upstream of the transcription start site of *Stat1*. BMDMs were pretreated with GPI or 6AN for 30 min prior to stimulation with IFN-γ/LPS for 24 h, RARβ enrichment around the promoter of *Stat1* were analyzed by ChIP-PCR and IgG was used as a negative control. ChIP-qPCR were used to *Stat1* quantitative detection and total genomic DNA was used as input. Data are presented as mean ± SEM of *n* = 3 biologically independent experiments (**a**, **b**, **e**, **g**, **j**, **l**, **n**, **o**, **q**) or *n* = 4 biologically independent experiments (**h**) or *n* = 12 from two independent experiments (**r**). *P* values were calculated using one-way ANOVA (**a**, **b**, **e**, **g**, **j**, **o**, **q**, and **r**) and two-tailed unpaired Student’s *t*-tests (**h**, **l**, and **n**).

Next, we investigated how the inflammatory macrophage phenotype was regulated by UDPG/P2Y_14_ signaling. STAT1 is known as a key transcription factor that mediates the macrophage inflammatory phenotype^33,34^. Indeed, *Stat1* knockdown downregulated inflammatory gene expression in IFN-γ/LPS-treated macrophages (Fig. 5a). Surprisingly, the expression of *Stat1* was markedly inhibited by *Pgm1*, *Ugp2*, *Pygl* or *P2ry*_*14*_ siRNA (Fig. 5b). However, the addition of exogenous UDPG could rescue *Stat1* expression in *Pgm1*-, *Ugp2*- or *Pygl*-knockdown IFN-γ/LPS-treated macrophages, but not in * P2ry*_*14*_-knockdown macrophages (Fig. 5b), suggesting that glycogen metabolism regulates *Stat1* expression via P2Y_14_ signaling. Several transcription factors including RARβ, ZNF-148 and IRF-1 have been reported to promote STAT1 expression^35–38^. Although RARβ, ZNF-148 and IRF-1 were highly expressed in inflammatory macrophages (Supplementary Fig. 5a), the blockade of the glycogen-PPP pathway by inhibitors or siRNAs only downregulated RARβ at both mRNA and protein levels, but did not influence ZNF-148 and IRF-1 (Fig. 5c–e and Supplementary Fig. 5b); and immunofluorescent staining showed a consistent result of the decreased nuclear location of RARβ (Fig. 5f). Intriguingly, knocking down P2Y_14_ receptor decreased RARβ levels and overexpressing P2Y_14_ receptor increased RARβ levels (Fig. 5g-i), suggesting that RARβ is regulated by UDPG/P2Y_14_ signaling. Indeed, addition of exogenous UDPG induced RARβ and STAT1 expression in macrophages (Fig. 5j, k). Also, overexpression of RARβ induced STAT1 and inflammatory gene expression (Fig. 5l-n and Supplementary Fig. 5c). Then, we used siRNA to knock down *Rarb*, and this resulted in the inhibition of the STAT1 and macrophage inflammatory phenotype (Fig. 5o-q and Supplementary Fig. 5d). Under this knockdown condition, the addition of exogenous UDPG could not rescue the inflammatory phenotype in the macrophages (Supplementary Fig. 5e). In addition, *Stat1* knockdown also invalidated the effect of the exogenous UDPG on the inflammatory phenotype in the macrophages (Supplementary Fig. 5e). Thus, the UDPG-P2Y_14_-RARβ axis was identified to regulate STAT1 expression. Moreover, analysis with the UCSC Genome Browser and JASPAR revealed the presence of multiple consensus cis-elements for RARβ binding on the promoter of *Stat1*, and a ChIP-PCR assay indicated that RARβ directly bound to the *Stat1* promoter (Fig. 5r), further confirming the above regulating axis. Despite the promoting effect of UDPG-P2Y_14_-RARβ on STAT1 in inflammatory macrophages, this axis seemed not to affect IL-4 stimulated macrophages, as evidenced by no induction of STAT1 by the addition of UDPG or the overexpression of P2Y_14_ or RARβ (Supplementary Fig. 5f, g).

**Fig. 6:**
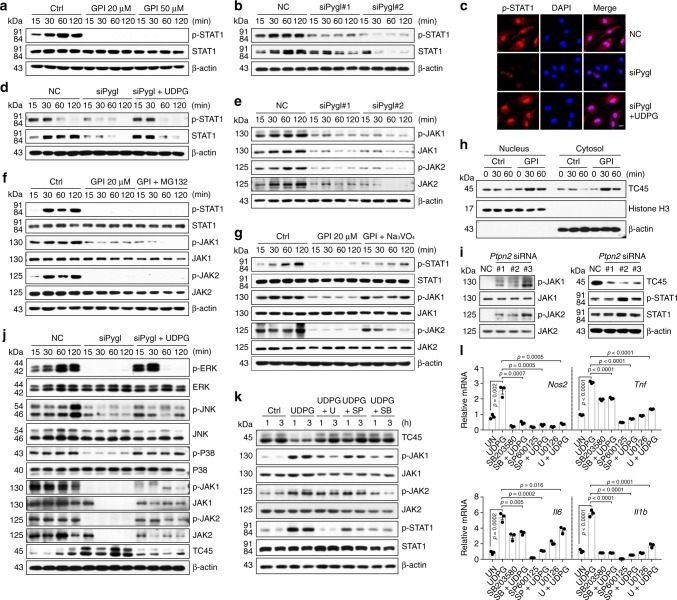
UDPG-P2Y_14_ pathway regulates STAT1 phosphorylation. **a**, **b** BMDMs were pretreated with GPI (20 or 50 μM) for 30 min or pretransfected with *Pygl* siRNA for 24 h prior to stimulation with IFN-γ/LPS, followed by western blot analysis of STAT1 and its phosphorylation from 15 to 120 min after stimulation. **c**, **d** *Pygl* siRNA transfected BMDMs were treated with IFN-γ/LPS ± UDPG, STAT1 and its phosphorylation was analyzed by two-photon confocal microscope, scale bar, 10 μm (**c**) and western blot (**d**). **e** BMDMs were pretransfected with *Pygl* siRNA for 24 h prior to stimulation with IFN-γ/LPS, followed by western blot analysis of JAK1 and JAK2 from 15 to 120 min after stimulation. **f**, **g** BMDMs were pretreated with GPI alone or combined with MG132 (**f**) or Na_3_VO_4_ (**g**) for 30 min prior to stimulation with IFN-γ/LPS, followed by western blot analysis of STAT1, JAK1, and JAK2 from 15 to 120 min after stimulation. **h** BMDMs were pretreated with GPI for 30 min prior to stimulation with IFN-γ/LPS, the cytoplasmic and nuclear protein fractions were blotted for TC45, β-actin (cytoplasmic marker) and Histone H3 (nuclear marker). **i** *Ptpn2* siRNA transfected BMDMs were stimulated with IFN-γ/LPS, STAT1, JAK1, JAK2, and TC45 were analyzed by western blot. **j** *Pygl* siRNA transfected BMDMs were stimulated with IFN-γ/LPS ± UDPG, ERK, JNK, P38, JAK1, JAK2, and TC45 were analyzed from 15 to 120 min after stimulation by western blot. **k**, **l** IFN-γ/LPS stimulated BMDMs were treated with UDPG alone or combined with U0126, SP600125 or SB203580 for 1 and 3 h, JAK1, JAK2, STAT1, and TC45 were analyzed by western blot (**k**). *Nos2, Tnf, Il6,* and *Il1b* expression was determined by real-time PCR (**l**). Data are presented as mean ± SEM of *n* = 3 biologically independent experiments. *P* values were calculated using one-way ANOVA.

In addition to STAT1 expression, we here also investigated whether UDPG-P2Y_14_ signaling regulated the activity of STAT1. STAT1 activation requires phosphorylation to form dimers, which are translocated into the nucleus where they exert their function^33,34^. We found that disruption of glycogenolysis by either GPI or *Pygl* siRNA markedly inhibited the phosphorylation of STAT1 at Tyr701 (Fig. 6a, b) as well as its nuclear translocation (Fig. 6c), which could be reversed by the addition of UDPG (Fig. 6c, d and Supplementary Fig. 6a), suggesting that the UDPG-P2Y_14_ signaling is required for STAT1 phosphorylation. Inflammatory macrophages use Janus kinases (JAK1/JAK2) to phosphorylate STAT1^39,40^. We found that *Pygl* siRNA or GPI treatment decreased the phosphorylation and the expression of JAK1 and JAK2 (Fig. 6e and Supplementary Fig. 6a). Such decreased phosphorylation was not mediated by the proteasome pathway, because the addition of proteasome inhibitor MG132 had no effect on JAK1/2 and STAT1 phosphorylation recovery (Fig. 6f). However, if we added Na_3_VO_4,_ the non-specific tyrosine phosphatase inhibitor, the above decreased phosphorylation of JAK1/2 and STAT1 was rescued (Fig. 6g and Supplementary Fig. 6b). T-cell protein tyrosine phosphatase 45 (TC45, encoded by *Ptpn2* gene), a nuclear protein tyrosine phosphatase, is the major enzyme that dephosphorylate JAK1/2 and STAT1^41–44^. We found that TC45 was upregulated in inflammatory macrophages after GPI treatment (Fig. 6h); and *Ptpn2* knockdown by siRNA recovered the decreased phosphorylation of JAK1/2 and STAT1 from the GPI treatment (Fig. 6i). Activation of MAP kinases ERK, JNK and P38 constitutes the core signaling pathway that mediates the inflammatory phenotype of macrophages^45,46^. Coincidently, the UDPG-P2Y_14_ signaling also induces the activation of MAP kinases^47–49^. We found that *Pygl* siRNA or GPI treatment markedly inhibited the phosphorylation of ERK1/2, JNK and P38 MAP kinases, however the addition of UDPG enhanced the phosphorylation of MAPKs (Fig. 6j and Supplementary Fig. 6a, c). Moreover, using ERK, JNK and P38 inhibitors (U0126, SP600125 and SB203580) to treat UDPG and IFN-γ/LPS-conditioned macrophages, we found that the expression of TC45 was markedly upregulated, concomitant with the decreased phosphorylation of JAK1/2 and STAT1 as well as the downregulation of inflammatory gene expression (Fig. 6k, l and Supplementary Fig. 6d). Thus, P2Y_14_ signaling may activate MAPKs so to downregulate TC45 expression. Together, these data suggest that the UDPG-P2Y_14_ pathway regulates both STAT1 expression and its activity.

### Macrophage glycogen metabolism regulates inflammation in vivo

**Fig. 7:**
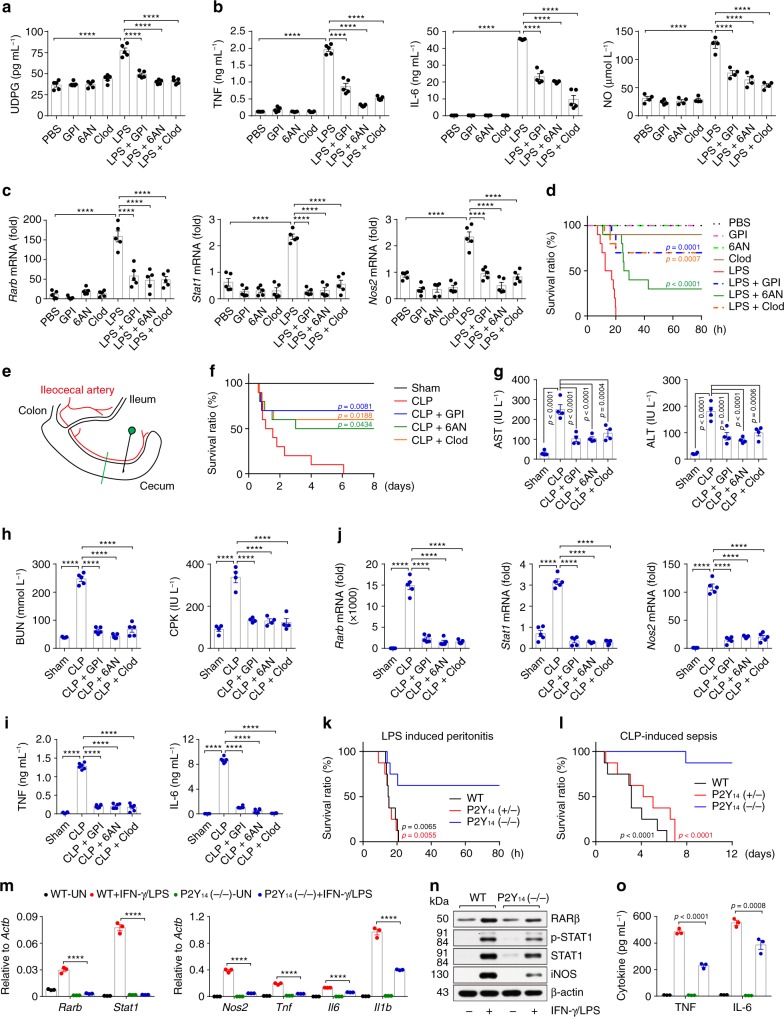
Macrophage glycogen metabolism regulates inflammation in vivo. **a**–**d** C57BL/6 J mice were treated with GPI, 6AN or clodronate liposomes, followed by i.p. injection of 20 μg g^−1^ body weight LPS. Four hours later, serum levels of UDPG were detected by ELISA, *n* = 5 mice per group (**a**). Serum levels of TNF and IL-6 (left and middle, *n* = 5 mice per group) and NO (right, *n* = 4 mice per group) were measured by ELISA (**b**). *Rarb*, *Stat1* and *Nos2* expression in peritoneal macrophages was determined by real-time PCR, *n* = 5 mice per group (**c**). The long-term survival of LPS-induced peritonitis was assessed, *n* = 10 mice per group, *p* values are presented relative to LPS group (**d**). **e** Schematic illustration showing the cecal ligation and puncture (CLP). **f**–**j** C57BL/6J mice were treated with GPI, 6AN or clodronate liposomes, followed by CLP procedure, the long-term survival of CLP-induced sepsis was recorded, *n* = 10 mice per group, *p* values are presented relative to CLP group (**f**). Serum levels of transaminase AST and ALT (**g**, *n* = 4 mice per group), BUN (**h**, left, *n* = 5 mice per group), CPK (**h**, right, *n* = 4 mice per group), TNF and IL-6 (**i**, *n* = 6 mice per group) were determined by ELISA. Peritoneal macrophages were isolated and *Rarb*, *Stat1* and *Nos2* expression was determined by real-time PCR, *n* = 5 mice per group (**j**). **k**, **l** Wide type (WT), P2Y_14_ (+/−) or P2Y_14_ (−/−) mice were injected intraperitoneally with 20 μg g^−1^ body weight LPS or were subjected to a sham or CLP procedure (*n* = 8 mice per group), the long-term survival was recorded, *p* values are presented relative to P2Y_14_ (−/−) group. **m**–**o** BMDMs from WT or P2Y_14_ (−/−) mice were stimulated with IFN-γ/LPS for 24 or 36 h, *Rarb*, *Stat1*, *Nos2*, *Tnf*, *Il6,* and *Il1b* expression was determined by real-time PCR, *n* = 3 mice per group (**m**), RARβ, STAT1, iNOS, TNF, and IL-6 expression was determined by western blot (**n**) and ELISA, *n* = 6 mice per group (**o**). Unless otherwise specified, *n* = 3 independent experiments were performed. Data are presented as mean ± SEM. *P* values were calculated using one-way ANOVA (**a**–**c**, **g**–**j**, **m** and **o**) and two-sided log-rank (Mantel-Cox) test (**d**, **f**, **k** and **l**). ****p < 0.0001.

Next, we validated the in vivo significance of the above elucidated glycogen metabolism in macrophages. In the LPS-induced acute peritonitis mouse model, an increased UDPG level was found, concomitant with the expression of inflammatory cytokines (TNF and IL-6) as well as NO (Fig. 7a, b); however, GPI or 6AN treatment resulted in decreased UDPG levels and markedly suppressed expression of inflammatory genes (Fig. 7a, b). Meanwhile, the depletion of peritoneal macrophages by clodronate liposomes (Clod) also led to the decrease of the inflammatory cytokines and NO in the above LPS-treated mice (Fig. 7a, b). Next, we isolated peritoneal macrophages from the GPI- or 6AN-treated mice, which showed an accumulation of glycogen and a marked downregulation in the expression of *Rarb*, *Stat1* and *Nos2* (Supplementary Fig. 7a and Fig. 7c). Moreover, while all the untreated mice died from LPS-induced peritonitis, most of those who were treated with GPI, 6AN or clodronate liposomes survived (Fig. 7d). Here, we also treated the mice with the GPI plus Clod or 6AN plus Clod, however the combination did not show different levels of TNF and IL-6 in the serum (Supplementary Fig. 7b), suggesting that the glycogen metabolism and PPP activity in macrophages might be the relevant target of these inhibitors in vivo. Besides LPS-induced peritonitis, similar results were obtained in the ConA-induced hepatitis model, where the treatment with GPI, 6AN or clodronate liposomes inhibited inflammatory cytokines (TNF and IL-6), reduced liver damage, and decreased the mortality rate (Supplementary Fig. 7c–f). To further convince the role of glycogen metabolism in acute inflammatory disorders, a cecal ligation and puncture (CLP)-induced sepsis mouse model was used (Fig. 7e)^50–52^. Blocking glycogen metabolism by either GPI or 6AN effectively inhibited sepsis, as evidenced by (1) 80% mice were rescued from septic death (Fig. 7f); (2) CLP-induced organic damage (liver, kidney and heart) was relieved (Fig. 7g, h); and (3) the levels of released inflammatory cytokines such as TNF and IL-6 were reduced (Fig. 7i). Consistently, *Rarb*, *Stat1* and *Nos2* was downregulated in the peritoneal macrophages after GPI or 6AN treatment (Fig. 7j), implicating that P2Y_14_ signaling regulates the inflammatory responses in vivo. We thus used P2Y_14_-/- mice to confirm this, it was found that P2Y_14_ knockout effectively prevented the death of the mice with peritonitis or sepsis (Fig. 7k, l). In line with these results, macrophages isolated from P2Y_14_−/− mice downregulated RARβ, STAT1 and pro-inflammatory cytokine expression in response to IFN-γ/LPS stimulation (Fig. 7m-o). However, this pro-inflammatory gene downregulation could be rescued by RARβ or STAT1 overexpression (Supplementary Fig. 7g-i). Together, these results suggest that the glycogen metabolism in macrophages regulates inflammatory responses.

### Macrophage glycogen metabolism regulates sepsis in patients

**Fig. 8:**
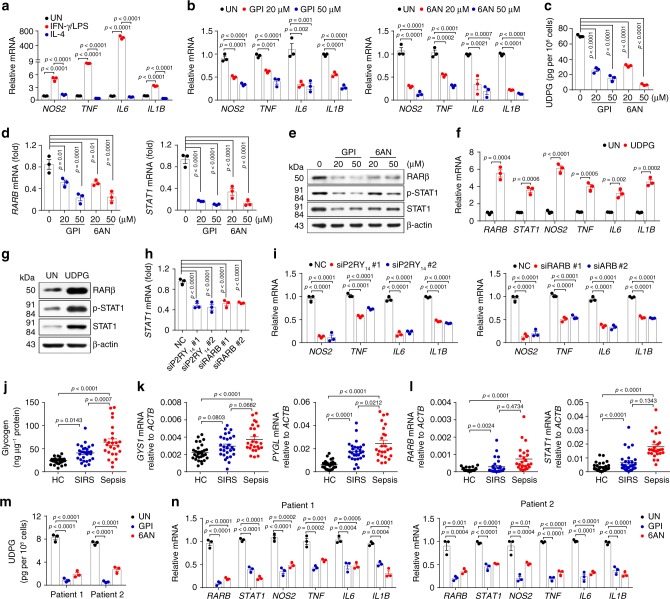
Macrophage glycogen metabolism regulates sepsis in patients. **a** THP-1 cells were cultured with PMA (100 ng mL^−1^) for 3 days and differentiated into macrophages, followed with IFN-γ/LPS or IL-4 stimulation for 24 h. *NOS2, TNF, IL6* and *IL1B* expression was determined by real-time PCR, *n* = 3 biologically independent experiments. **b**–**e** PMA incubated THP-1 cells were pretreated for 30 min with GPI or 6AN prior to stimulation with IFN-γ/LPS for 24 or 36 h, *NOS2, TNF, IL6,* and *IL1B* expression was determined by real-time PCR, *n* = 3 biologically independent experiments (**b**). UDPG in supernatants was determined by ELISA, *n* = 3 biologically independent experiments (**c**). RARβ and STAT1 expression was determined by real-time PCR, *n* = 3 biologically independent experiments (**d**) and western blot (**e**). **f**, **g** PMA and IFN-γ/LPS-stimulated THP-1 cells were treated with or without UDPG for 24 or 36 h, *RARB*, *STAT1*, *NOS2*, *TNF, IL6,* and *IL1B* expression was determined by real-time PCR, *n* = 3 biologically independent experiments (**f**), RARβ and STAT1 expression was determined by western blot (**g**). **h**, **i** *P2RY*_*14*_ or *RARB* siRNA transfected THP-1 cells were stimulated with IFN-γ/LPS for 24 h, *STAT1*, *NOS2*, *TNF*, *IL6,* and *IL1B* expression was determined by real-time PCR, *n* = 3 biologically independent experiments. **j**–**l** Blood samples from patients with sepsis (*n* = 25) and SIRS (*n* = 28) and healthy controls (*n* = 30) were collected. Intracellular glycogen levels in human peripheral blood CD14^+^ monocytes were determined by colorimetric assay (**j**). *GYS1*, *PYGL*, *RARB,* and *STAT1* expression was determined in human peripheral blood CD14^+^ monocytes by real-time PCR (**k**, **l**). **m**, **n** Two sepsis patients’ CD14^+^ monocytes were isolated and cultured with M-CSF (20 ng mL^−1^) for 4 days, and then treated with GPI or 6AN respectively. UDPG in supernatants was determined by ELISA, *n* = 3 biologically independent experiments (**m**) and *RARB*, *STAT1*, *NOS2*, *TNF*, *IL6,* and *IL1B* expression was determined by real-time PCR (**n**), *n* = 3 biologically independent experiments. Data are presented as mean ± SEM. *P* values were calculated using one-way ANOVA (**a**–**d** and **h–n**) and two-tailed unpaired Student’s *t*-tests (**f**).

To validate the above murine data in human inflammatory responses, we first repeated the results in the human monocytic THP-1 cells, the most widely used model for human macrophages^53^. Similarly, IFN-γ/LPS-treated THP-1 cells displayed the upregulation expression of inflammatory phenotypes *NOS2*, *TNF*, *IL6,* and *IL1B* (Fig. 8a), However, disruption of either glycogenolysis or PPP by inhibitors (GPI or 6AN) resulted in marked decrease of the expression of *NOS2*, *TNF*, *IL6,* and *IL1B* (Fig. 8b). In line with the subdued inflammatory phenotype, marked decrease of UDPG levels were also observed, concomitant with the downregulation of *RARB* and *STAT1* expression (Fig. 8c-e). By contrast, the addition of exogenous UDPG led to the upregulation of inflammatory gene expression as well as the RARβ and STAT1 expression (Fig. 8f, g). In addition, treatment with *P2RY*_*14*_ or *RARB* siRNA also downregulated *STAT1* expression and inflammatory gene expression (Fig. 8h, i and Supplementary Fig. 8a, b). These data suggest that glycogen metabolism regulates inflammatory phenotype in human macrophages.

Excessive activation of innate immune cells may lead to SIRS or even sepsis^1–3,54^. Given the crucial role of macrophages in human inflammatory conditions and in the above-described sepsis mouse model, we here further investigated whether glycogen metabolism was involved in human SIRS or sepsis. For this purpose, blood samples from patients with sepsis (*n* = 25) or SIRS (*n* = 28) and healthy controls (*n* = 30) were collected (Supplementary Table 1). We found that glycogen levels in CD14^+^ monocytes were higher in SIRS and sepsis patients compared to healthy donors (Fig. 8j). In accordance with this, the expression of glycogenic and glycogenolytic enzymes was also upregulated (Fig. 8k), concomitant with increased expression of *RARB* and *STAT1* (Fig. 8l). When we cultured the CD14^+^ monocytes and treated them with GPI or 6AN, we found that blocking glycogenolysis or PPP led to the decrease of UDPG levels, downregulation of inflammatory gene expression as well as *RARB* and *STAT1* genes (Fig. 8m, n). Together, these results suggest that glycogen metabolism of human macrophages regulates inflammatory responses in septic patients.

## Discussion

Remodeled metabolism is known to be vital for a macrophage-mediated inflammatory response, but the underlying mechanism remains largely unclear, especially for the regulation of inflammatory gene expression by the altered metabolism. In this study, we provide evidence that macrophages mobilize glycogen metabolism, which governs macrophage-mediated inflammatory response. On one hand, glucose through the PPP via glycogenesis and glycogenolysis, thus providing antioxidative NADPH for the survival of activated inflammatory macrophages; on the other hand, glycogen metabolism produces an intermediate metabolite UDPG which triggers the P2Y_14_ signaling pathway, which then regulates the key inflammatory transcription factor STAT1 expression and activity.

Glycogen, the long-term reservoir of glucose, is known to be primarily produced by liver and muscle cells, thus providing energy for cells and maintaining blood sugar homeostasis of the body^24^. Notwithstanding this original understanding, the physio-pathological role of glycogen metabolism may be more complex. We previously reported that CD8^+^ memory T cells use a glycogen metabolic program to regulate memory formation and maintenance^17^. In the present study, we further show that an active glycogen metabolism governs the inflammatory phenotype of macrophages. In these macrophages, the end product of glycogen metabolism, G6P, is not channeled to glycolysis but to the PPP. Previously, an active PPP was observed in inflammatory macrophages^11,13,55^. However, those studies considered that the PPP was directly branched from glycolysis and used glycolysis-derived G6P. Nevertheless, by conducting ^13^C tracing assay, here we provide clear evidence that the G6P is not directly derived from glycolysis but from glycogenolysis. Following glucose phosphorylation by hexokinase, the formed G6P is first used in glycogenesis. Then, the glycogen is degraded to produce G6P again and the latter is channeled through the PPP. Identification of this metabolic pathway undoubtedly provides new insight into how glucose metabolism regulates macrophage phenotype, but raises the question that why macrophages use G6P to synthesize glycogen and then degrade glycogen to produce G6P, an overt futile glycogen synthesis/degradation cycle. This might be due to the distinctive compartmentalization of the involved enzymes. Another explanation is that glycogen metabolism triggers the UDPG-P2Y_14_ signaling pathway that regulates inflammatory gene expression in macrophages. UDP-glucose (UDPG) and other UDP-sugars are essential for glycosylation. Following the synthesis in the cytosol, UDPG is translocated to the ER/Golgi via ER/Golgi-resident SLC35 transporters, where UDPG is used for glycosylation. On the other hand, UDPG in the ER/Golgi lumen can also be released as cargo to the extracellular space via the constitutive secretory pathway^30,31^. This released UDPG can bind with the P2Y_14_ receptor via an autocrine or paracrine pattern.

P2Y_14_ is a purinergic G-protein coupled receptor, which can be expressed by immune cells including macrophages^56,57^. Notably, UDPG can act as the agonist to activate P2Y_14_ signaling, which may include phosphatidylinositol 3-kinase-γ, GPCR kinases 2 and 3, phospholipase C and MAPKs^47–49,58^. In the present study, we further identify that UDPG-P2Y_14_ signaling regulates the expression and phosphorylation of STAT1 via upregulating transcription factor retinoic acid receptor RARβ and downregulating tyrosine phosphatase TC45. In this study, we did not elucidate how RARβ was regulated through the UDPG-P2Y_14_ signaling. RARs, composed of three subtypes α, β and γ, act as ligand-activated vfactors through dimerizing with retinoid X receptors (RXR) and binding to retinoic acid response elements (RARE) in the promoter region of target genes. Intriguingly, RARβ itself can be one of the target genes^59^. More recently, a study showed that RARβ was upregulated by the MAPK (ERK1/2 and P38) signaling, which was consistent with an earlier report that nerve growth factor-activated Ras signal pathway for RARβ upregulation^60^. Notably, P2Y_14_ signaling also activates MAPK. Thus, P2Y_14_ signaling might regulate RARβ levels through the MAPK pathway. All in all, the elucidation of the UDPG-P2Y_14_ signaling provides an example insight into how glucose metabolism is involved in the signaling pathway of cells. In addition to inflammatory macrophages, other immune cell type(s) might also mobilize this signaling pathway to respond innate stimulation. Neutrophils are some similar to macrophages in that they have the common progenitors and neutrophils also have the phagocytotic function. Whether neutrophils use glycogen metabolism to trigger the UDPG-P2Y_14_ signaling is worthy of further investigation.

Inflammatory macrophages play a crucial role in mediating acute immune responses that cause pathological tissue damage in various inflammatory diseases^1–3,54,61^. The glycogen-PPP and UDPG-P2Y_14_ pathways identified in this study may have important clinical significance and provide potential therapeutic targets to block acute inflammatory responses. Indeed, in murine models of both acute peritonitis and liver damage, blocking glycogen-PPP metabolic or UDPG-P2Y_14_ signaling pathway effectively inhibits the inflammatory response and averts certain death for the mice. More importantly, such treatment also produces an efficacious outcome in the mouse sepsis model. Sepsis is a serious clinical issue, causing millions of deaths worldwide each year^61–65^. Analysis of clinical samples of SIRS/Sepsis patients also indicates that macrophages activate glycogen-PPP and UDPG-P2Y_14_ pathways to polarize an inflammatory phenotype, uncovering a potential strategy against clinical sepsis. Upon activation, macrophages can be polarized to an inflammatory or anti-inflammatory phenotype. Although the glycogen-PPP or UDPG-P2Y_14_ pathway is required for an inflammatory phenotype, using siRNA (*Pgm1*, *Ugp2*, *Pygl*, *G6pdx* or *P2ry*_*14*_) to block the glycogen-PPP or UDPG-P2Y_14_ pathway did not switch IFN-γ/LPS-stimulated macrophage development toward anti-inflammatory phenotype such as increased levels of arginase 1 and IL-10 (Supplementary Fig. 9). In addition to sepsis, these findings have potential value to break immunological tolerance in cancer. For instance, it is possible for us to use glycogen synthase activator to reset tumor-associated macrophages toward inflammatory phenotype through the regulation of glycogen metabolism.

**Fig. 9:**
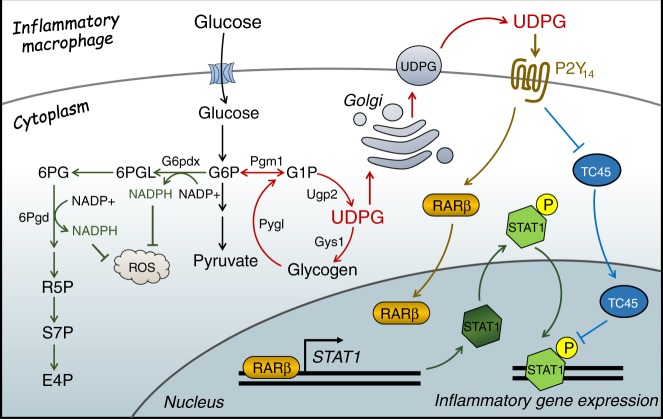
Glycogen metabolism regulates macrophages. In inflammatory macrophages, glycogen is synthesized and then channeled through glycogenolysis to generate G6P and further through the pentose phosphate pathway to yield abundant NADPH, ensuring high levels of reduced glutathione for inflammatory macrophage survival. Glycogen metabolism also increases UDPG levels and the receptor P2Y_14_ in macrophages. The UDPG/P2Y_14_ signaling pathway not only upregulates the expression of STAT1 via activating RARβ but also promotes STAT1 phosphorylation by downregulating phosphatase TC45.

In summary, the data in this study clearly show that macrophages, by virtue of their mobilizing glycogen metabolism, are polarized to an inflammatory phenotype. This metabolic pathway not only enhance the PPP that provides NADPH for antioxidation and cell survival, but also triggers the UDPG-P2Y_14_ signaling pathway to promote the expression and activity of the key inflammatory transcription factor STAT1 (Fig. 9). Therefore, we identify a previously unknown key metabolic pathway that fundamentally regulates the inflammatory phenotype of macrophages, which provides novel strategies against inflammatory diseases.

## Methods

### Mice

Female wild-type C57BL/6J mice with 5–7-weeks old were purchased from the Center of Medical Experimental Animals of Hubei Province (Wuhan, China) and P2Y_14_^+/−^ and P2Y_14_^−/−^ mice were purchased from the Cyagen Biosciences Inc. For studies, all mice were kept under SPF conditions at the Animal Care and Use Committee of Tongji Medical College. All animal experiments were conducted in accordance with a protocol approved by the Animal Care and Use Committee of Tongji Medical College (ethics number: S1891).

### Cells

Human monocytic THP-1 cells were purchased from the China Center for Type Culture Collection (Wuhan, China) and cultured in complete RPMI-1640 medium containing 10% fetal bovine serum, 10 mM glucose, 2 mM L-glutamine and 100 U mL^−1^ penicillin-streptomycin. THP-1 cells were differentiated into M0 macrophages by incubation with 100 ng mL^−1^ phorbol 12-myristate 13-acetate (PMA) (P8139, Sigma-Aldrich) for 72 h. Once the cells were adherent, they were transferred to PMA-free media to obtain resting macrophages. These cells were incubated with 20 ng mL^−1^ human macrophage colony-stimulating factor (M-CSF) (300-25, PeproTech) and then co-stimulated with 100 ng mL^−1^ LPS (L2630, Sigma-Aldrich) plus 20 ng mL^−1^ IFN-γ (300-02, PeproTech) or stimulated with 10 ng mL^−1^ IL-4 (200-04, PeproTech) for 24 h to generating inflammatory or anti-inflammatory macrophages.

### Human samples

Human peripheral blood was obtained from patients at ICU of Union Hospital (Wuhan, China). Ethical permission was granted by the Ethics Committee of the Huazhong University of Science and Technology. All patients provided written informed consent to participate in the study.

### Reagents

GPI (PZ0189), 6AN (A68203), UDPG (U4625), ConA (L7647), PPTN trifluoroacetate salt (SML1809), MG-132 (M8699), Na_3_VO_4_ (S6508), SB203580 (S8307), SP600125 (S5567), U0126 (19-147) were purchased from Sigma-Aldrich. Clodronate liposomes was purchased from Liposoma. Glucose (GO) Assay Kit (GAGO20) and Periodic Acid-Schiff (PAS) Assay Kit (395B) were purchased from Sigma-Aldrich. The primary antibodies were purchased from Cell Signaling Technology: anti-iNOS (D6B6S#13120, 1:1000), anti-phospho-STAT1-Tyr701 (58D6#9167, 1:1000), anti-STAT1 (9H2#9176, 1:1000), anti-JAK1 (D1T6W#50996, 1:1000), anti-phospho-ERK-T202/Y204 (194G2#4377S, 1:1000), anti-ERK (137F5#4695S, 1:1000), anti-phospho-JNK-T183/Y185 (9251S, 1:1000), anti-JNK (9252S, 1:1000), anti-phospho-P38-Thr180/Tyr182 (3D7#9215, 1:1000), anti-P38 (9212S, 1:1000), anti-Fbp1 (D2T7F, 1:1000), anti-G6pdx (#8866, 1:1000), anti-Gys1 (3893S, 1:1000), anti-TC45 (D7T7D#58935, 1:1000) and anti-IRF-1 (D5E4#8478, 1:1000). The following primary antibodies were purchased from Abcam: anti-Pgm1 (ab192876, 1:1000), anti-Ugp2 (ab154817, 1:1000), anti-Pygl (ab190243, 1:1000), anti-Pygm (ab88078, 1:1000), anti-P2Y_14_ (ab136264, 1:1000), anti-G6pase (ab83690, 1:1000) and anti-Histone H3 (ab8284, 1:1000). Anti-phospho-JAK1-Y1022/1023 (YP0154, 1:1000), anti-phospho-JAK2-Tyr570 (YP0306, 1:1000) and anti-JAK2 (YT2428, 1:1000) were purchased from Immunoway. Anti-Pck1 (Z6754-Z-AP, 1:1000) was purchased from Proteintech. Anti-6Pgd (A7710, 1:1000), anti-HK1 (A1054, 1:1000), anti-HK2 (A0994, 1:1000) and anti-HK3 (A8428, 1:1000) were purchased from ABclonal. Anti-ZNF148 (QC7801, 1:1000), anti-RARβ (310315, 1:1000) and anti-β-actin (A1978, 1:10000) were purchased from Sigma-Aldrich. The secondary antibody goat anti-rabbit IgG Dylight®594 (ab96885, 1:400) was purchased from Abcam, HRP-goat anti-rabbit and HRP-goat anti-mouse (1:10000) were purchased from EARTH.

### Preparation of mouse macrophages

Bone marrow cells isolated from C57BL/6J mice were cultured for 5 days in the complete RPMI-1640 medium containing 20 ng mL^−1^ recombinant mouse M-CSF (315-02, PeproTech), 10% fetal bovine serum, 10 mM glucose, 2 mM L-glutamine and 100 U mL^−1^ penicillin-streptomycin. On day 6, macrophages were co-stimulated with 100 ng mL^−1^ LPS plus 20 ng mL^−1^ IFN-γ (315-05, Sigma-Aldrich) or stimulated with 10 ng mL^−1^ IL-4 (214-14, Sigma-Aldrich) for 24 h to generating inflammatory or anti-inflammatory macrophages. Mouse peritoneal macrophages were harvested by peritoneal lavage. Cold PBS was injected into the peritoneal cavity and extracted after gentle agitation. The peritoneal cell suspension was centrifuged at 1300 rpm, and the cell pellet was mixed with 2 mL Red blood cell lysis buffer for 5 min at room temperature. After washing, the cells were cultured on six-well plate for 3 h. The adhesion cells were collected as peritoneal macrophages.

### Isolation of human monocytes

Human peripheral blood mononuclear cells were isolated from human peripheral blood using density gradient separation. Monocytes were purified by human CD14 Micro-Beads (130-050-201, MACS) and then cultured in complete RPMI 1640 medium containing 20 ng mL^−1^ recombinant human M-CSF for the induction of macrophages. Seven days later, human macrophages were harvested and stimulated with 100 ng mL^−1^ LPS plus 20 ng mL^−1^ human IFN-γ or 10 ng mL^−1^ human IL-4 for inflammatory and anti-inflammatory macrophages.

### Electron microscopy

The untreated, IFN-γ/LPS or IL-4-treated macrophages were washed with PBS three times, then fixed in 2.5% glutaraldehyde in 0.1 M PBS and processed for routine electron microscopy as described previously^17^. Briefly, the samples were post fixed in 1% osmium tetroxide (OsO_4_) for 100 min at room temperature, and rinsed with distilled water three times. The pellets were then dehydrated in a graded ethanol series, treated with propylene oxide and embedded with Spurr’s epoxy resin. Cut sections were stained with uranyl acetate and lead citrate, and then imaged using a JEM1010 electron microscope (JEOL).

### Glucose consumption

The untreated, IFN-γ/LPS or IL-4-treated macrophages after complete medium exchange, a cell-free control group were set. Following a 24-h incubation, the supernatants were collected and glucose concentration was measured using Glucose Assay Kit according to the manufacturer’s instructions. Cells were lysed for protein quantification using a BCA Protein Assay Kit (23227, Thermo Fisher). Glucose consumption rate was determined as the glucose concentration in the supernatants minus that of the cell-free control group, and normalized to total protein levels.

### Metabolite analysis

For ^13^C tracing experiments, bone marrow cells were cultured in 5% CO_2_ conditions and differentially cultured into macrophages. On day-6 of culture, cells were washed, then cultured with [U6]-^13^C glucose (389374, Sigma-Aldrich), [U3]-^13^C pyruvate (490717, Sigma-Aldrich) or [U2]-^13^C acetate (282014, Sigma-Aldrich) for 24 h. Cells were washed twice in saline and lysed in extraction solvent (80% methanol/water) for 30 min at −80 °C. After centrifugation at 13,000 × *g*, 10 min at 4 °C, supernatant extracts were analyzed by LC-MS/MS as described previously. Briefly, liquid chromatography was performed using a HPLC (Ultimate 3000 UHPLC) system (Thermo Fisher) equipped with An X bridge amide column (100 × 2.1 mm i.d., 3.5 μm; Waters). The column temperature was maintained at 10 °C. The mobile phase A is 20 mM ammonium acetate and 15 mM ammonium hydroxide in water with 3% acetonitrile, pH 9.0, and mobile phase B is acetonitrile. The linear gradient is as follows: 0 min, 85% B; 1.5 min, 85% B, 5.5 min, 30% B; 8 min, 30% B, 10 min, 85% B, and 12 min, 85% B. The flow rate was 0.2 mL min^−1^. Sample volumes of 5 μl were injected for LC-MS/MS analysis. LC-MS/MS analysis was performed on a Q-exactive mass spectrometer (Thermo Fisher) equipped with a HESI probe, and the relevant parameters are as listed: heater temperature, 120 °C; sheath gas, 30; auxiliary gas, 10; sweep gas, 3; spray voltage, 2.5 kV for the negative mode. A full scan ranges from 80 to 350 (*mz*^−1^) was used. The resolution was set at 70,000. Data were captured using the Xcalibur™ software, version:3.0 (Thermo Fisher) and quantified by integrating the area underneath the curve of each compound using Xcalibur Qual browser (Thermo Fisher). Each metabolite’s accurate mass ion and subsequent isotopic ions were extracted (EIC) using a 10 ppm window.

### The level of UDPG, glycogen, and LDH

The level of UDPG, glycogen or LDH was measured by UDPG Detection Kit (mouse RY-03799, human RY-11791, Shanghai RunYu Biotech), Glycogen Assay Kit (KA0861, Abnova) or CytoTox 96 Non-Radioactive Cytotoxicity Assay (G1780, Promega), respectively, according to the manufacturer’s Instructions.

### NADPH/NADP+ and GSH/GSSG assay

The NADPH/NADP+ ratio was determined with the NADP/NADPH Quantification Colorimetric Kit (KA1663, Abnova). Measurements were performed according to the manufacturer’s instructions. The GSH/GSSG ratio was measured by LC/MS/MS.

### Detection of ROS

ROS levels were measured using CellROX™ Green Flow Cytometry Assay Kit (C10444, Invitrogen). Cells were loaded with 500 nM CellROX Green for 30 min at 37 °C, protected from light. Cells were washed and scraped in PBS and immediately analyzed by flow cytometry, using 488 nm excitation for the CellROX Green.

### Plasmid constructs and transfection

Recombinant vectors encoding murine *P2ry*_*14*_, *Stat1* and *Rarb* were constructed by PCR-based amplification from cDNA of BMC and then were subcloned into the GV230 (*P2ry*_*14*_), pcDNA3.1(+) × Flag (*Stat1*) eukaryotic expression vectors or GV287 (*Rarb*) lentivirus expression vector. All constructs were confirmed by DNA sequencing. Plasmids were transiently transfected into BMM with Lipofectamine 2000 transfection reagent (11668019, Invitrogen).

### Gene silencing experiments

siRNAs targeting mouse *Hk1*, *Hk2*, *Hk3*, *Pygl*, *P2ry*_*14*_, *Ugp2*, *Stat1*, *Gys1*, *G6pdx*, *Rarb*, *Pgm1*, *Ptpn2* and human *P2RY*_*14*_, *RARB* and negative control siRNAs (NC) were purchased from RiboBio (Guangzhou, China). siRNA (50 nM) was transfected into BMC using Lipofectamine™ RNAiMAX Transfection Reagent (13778150, Invitrogen) according to the manufacturer’s instruction. The siRNA sequences are shown as Supplementary Table 2.

### Real-time PCR

Total RNA extraction was prepared with TRIzol reagent (15596026, Invitrogen) and the cDNAs were generated by ReverTra Ace qPCR RT Kit (FSQ-101, Toyobo). Real-time PCR was performed for all genes with primers on a Bio-Rad CFX Connect and the data was captured using Bio-Rad CFX Manager 2.0 software. The expression of mRNA for genes of interest was normalized to *Actb* (Mus) *or ACTB* (Homo). The entire procedure was repeated in at least three biologically independent samples. The primer sequences are shown as Supplementary Table 3.

### Western blot analysis

Cell lysates and pre-stained molecular weight marker were separated by SDS-PAGE, followed by transfer onto nitrocellulose membranes. The membranes were blocked in TBST (Tris-buffered saline with 0.5% Tween 20) containing 5% bull serum albumin (BSA) and probed with specific antibodies overnight at 4 °C. The membranes were washed three times and incubated with horseradish peroxidase-conjugated secondary antibodies. The immunoreactivity was visualized by enhanced chemiluminescence according to the manufacturer’s protocol (ECL Kit, 34577, Pierce).

### Two-photon confocal microscopy

For intracellular staining, bone marrow cells were fixed in 2% paraformaldehyde for 10 min at room temperature, permeabilized with 100 μM digitonin and blocking with 1% BSA for 1 h at 25 °C. Samples were incubated with primary antibody (in PBST and 1% BSA) overnight at 4 °C. Cells were washed three times in PBS and incubated with secondary antibodies for 1 h at room temperature. Nuclei were stained in DAPI solution (1 μg mL^−1^). The confocal images were observed under confocal microscope (Leica SP8) and captured using LAS X Life Science Software, version: 3.0.16120.2 (Buffalo Grove, IL 60089 United States).

### ELISA

TNF (900-K54, PeproTech) and IL-6 (900-K50, PeproTech) production in the supernatants were quantified by ELISA kits according to the manufacturer’s protocol. Alanine amino transferase (ALT), aspartate amino transferase (AST), blood urea nitrogen (BUN), creatinephosphokinase (CPK) and serum concentrations were measured by ELISA according to manufacturer instructions.

### ChIP-qPCR

Chromatin immunoprecipitation (ChIP) Assay Kit (53009, Active Motif) was utilized to examine the binding of RARβ to the *Stat1* promoter. Macrophages treated with GPI or 6AN fixed with 1% formaldehyde on ice to cross-link the proteins bound to the chromatin DNA. After washing, the chromatin DNA was sheared by enzymatic force to produce DNA fragments of around 200–1000 bp. The same amounts of sheared DNA were used for immunoprecipitation with a RARβ antibody (SAB4502581, Sigma-Aldrich) or an equal amount of pre-immune rabbit IgG (Gene Tex). The immunoprecipitate then was incubated with protein G Magnetic Beads, and the antibody-protein G Magnetic Beads complex was collected for subsequent reverse cross-linking. The same amount of sheared DNA without antibody precipitation was processed for reverse cross-linking and served as input control. DNA recovered from reverse cross-linking was used for PCR. PCR was performed with primers for the *Stat1* promoter (forward, 5′-GACCAAG CAAGAAAAGCCATCAT-3′ and reverse, 5′-CTCGAGCCTCGGTTGGTCTA-3′) flanking the RARβ binding site at 59 °C for 36 cycles.

### Animal studies

In the LPS-induced acute peritonitis mouse model, C57BL/6J mice were treated with GPI (10 μg g^−1^, i.p., 12 and 4 h before LPS injection), 6AN (15 μg g^−1^, i.p., 12 and 4 h before LPS injection) or clodronate liposomes (Clod, 10 μL g^−1^, i.p., 36 and 4 h before- and 36 h after- LPS injection), followed by i.p. injection of 20 μg g^−1^ body weight LPS. In the ConA-induced hepatitis model, C57BL/6J mice were treated with GPI (10 μg g^−1^, i.v., 12 and 4 h before ConA injection), 6AN (15 μg g^−1^, i.v., 12 and 4 h before ConA injection) or clodronate liposomes (Clod, 10 μL g^−1^, i.p., 36 and 4 h before- and 36 h after- ConA injection), followed by i.v. injection of 15 μg g^−1^ body weight ConA. In the cecal ligation and puncture (CLP)-induced sepsis mouse model, C57BL/6J mice were treated with GPI (10 μg g^−1^, i.p., 12 and 4 h before CLP procedure), 6AN (15 μg g^−1^, i.p., 12 and 4 h before CLP procedure) or clodronate liposomes (10 μL g^−1^, i.p., 36 and 4 h before- and 36 h after- CLP procedure), followed by CLP procedure.

### Statistics

All experiments were performed at least three times. Results were expressed as mean ± SEM and analyzed by two-tailed unpaired Student *t* test or one-way ANOVA. In all tests, *p* values of <0.05 were considered statistically significant. The analysis was conducted using the GraphPad Prism 8.0 software.

### Reporting summary

Further information on research design is available in the Nature Research Reporting Summary linked to this article.

## Supplementary information

Supplementary Information

Reporting Summary

## Data Availability

The authors declare that the data supporting the findings of this study are available within the article and its Supplementary Information files and from the corresponding author on reasonable request. The raw data for the figures and supplementary figures are presented in the Source Data file.

## Source Data

Source Data
