# Supplementary material for: Glycogen metabolism regulates macrophage-mediated acute inflammatory responses
Source: Nat Commun. 2020 Apr 14;11:1769. doi: 10.1038/s41467-020-15636-8 (PMC7156451; doi:10.1038/s41467-020-15636-8)

Supplemental information

Glycogen metabolism regulates macrophage-mediated acute inflammatory responses

Ma et al.

Supplementary Figures

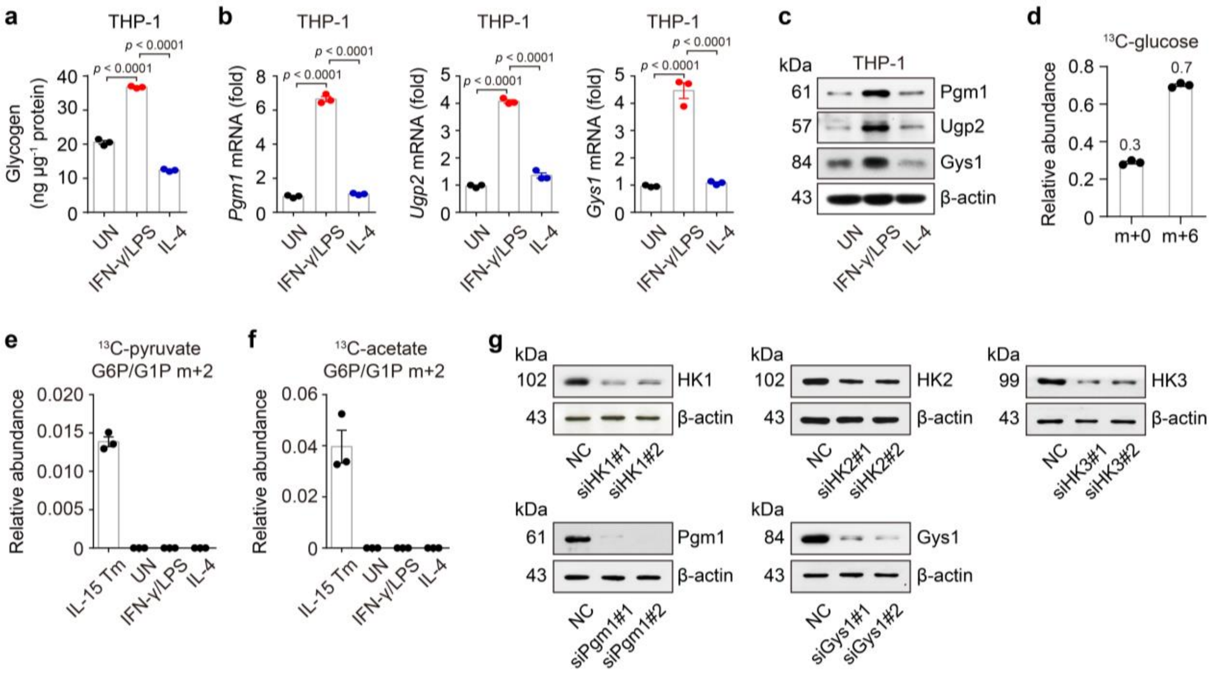

Supplementary Figure 1. Glycogen is Effectively Synthesized in Inflammatory Macrophages, Related to Fig. 1.

a THP-1 cells were cultured with PMA (100 ng mL^{-1}) for 3 days and differentiated into macrophages, followed with IFN-γ/LPS or IL-4 stimulation for 24 hr. Intracellular glycogen levels in untreated, IFN-γ/LPS or IL-4 treated cells were detected by colorimetric assay. **b, c** Pgm1, Ugp2 and Gys1 expression in untreated, IFN-γ/LPS or IL-4 treated THP-1 cells were determined by real-time PCR (**b**) and western blot (**c**). **d** BMDMs cultured and differentiated in ^{13}C-glucose medium for 5 days and then stimulated with IFN-γ/LPS for 24 hours, followed by the treatment with hydrochloric acid, leading to the degradation of polymer glycogen into monomer glucose. The released ^{13}C-labeled glucose was determined by LC-MS/MS. **e, f** BMDMs differentiated in normal ^{12}C-glucose were stimulated with IFN-γ/LPS or IL-4 for 6 hr and switched to ^{13}C-pyruvate (**e**) or ^{13}C-acetate (**f**) for 6 hr, LC-MS/MS was performed for m+2-labelled G6P/G1P. IL-15 induced memory T cells as a positive control. **g** BMDMs were pretransfected with siRNA (HK1/2/3, Pgm1 or Gys1) for 24 hr prior to stimulation with IFN-γ/LPS for 36 hr, HK1/2/3, Pgm1 or Gys1 expression was examined by western blot. Unless otherwise specified, n = 3 biologically independent experiments were performed. Data are presented as mean ± SEM. *P* values were calculated using one-way ANOVA.

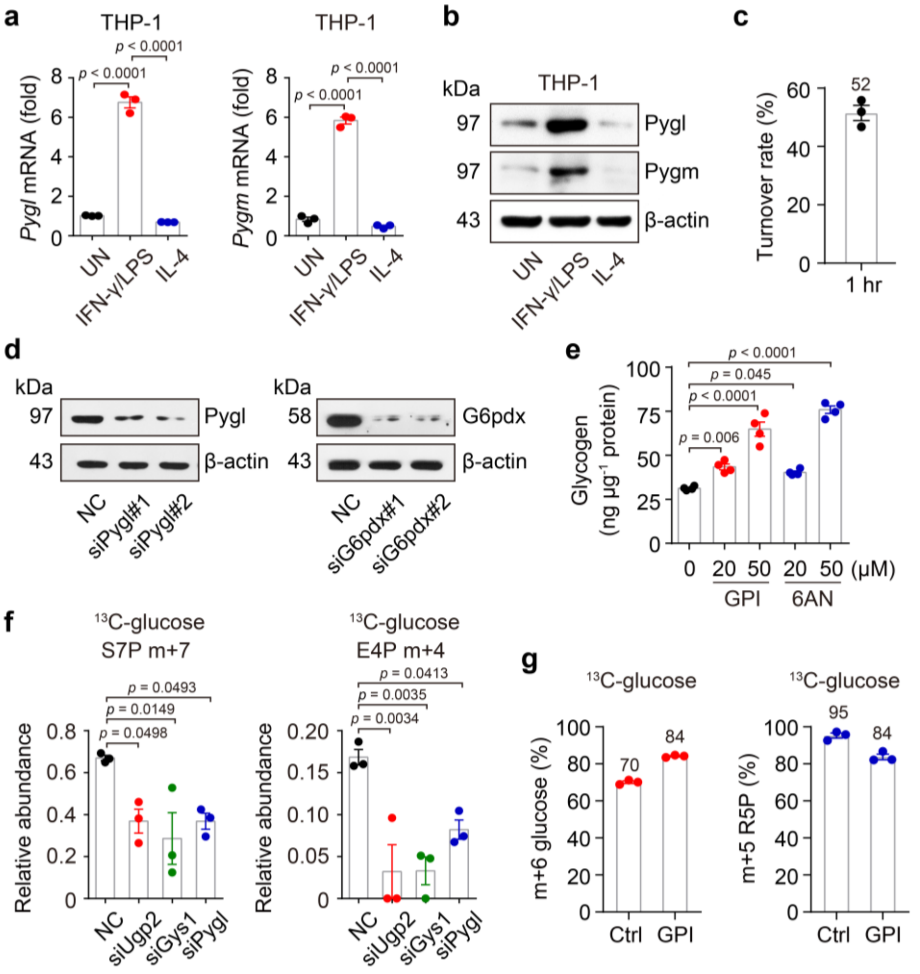

Supplementary Figure 2. Glycogenolysis-derived G6P is Channeled to the PPP, Related to Fig. 2. **a, b** THP-1 cells were cultured with PMA (100 ng mL^{-1}) for 3 days and differentiated into macrophages, followed with IFN-γ/LPS or IL-4 stimulation for 24 or 36 hr. Pygl and Pygm expression in untreated, IFN-γ/LPS or IL-4 treated THP-1 cells were determined by real-time PCR (**a**) and western blot (**b**). **c** BMDMs cultured and differentiated in ^{13}C-glucose, were stimulated with IFN-γ/LPS for 24 hours and then switched to ^{12}C-glucose for different time (0 or 1 hr). The cells were collected and treated with hydrochloric acid for the analysis of ^{13}C-labeled monomer glucose. Turnover rate = (m6-0hr minus m6-1hr) / m6-0hr. **d** Murine BMDMs were pretransfected with siRNA (Pygl or G6pdx) for 24 hr prior to stimulation with IFN-γ/LPS for 36 hr, Pygl or G6pdx expression was examined by western blot. **e** Murine BMDMs were pretreated with GPI or 6AN for 30 min prior to stimulation with IFN-γ/LPS for 24 hr, intracellular glycogen levels were detected by colorimetric assay. **f** Murine BMDMs were pretransfected with siRNA (Ugp2, Gys1 or Pygl) for 24 hr prior to stimulation with IFN-γ/LPS for 4 hr and m+7-labelled S7P and m+4-labelled E4P were analyzed by LC-MS/MS. **g** Bone marrow cells were cultured in ^{13}C-glucose medium and induced to differentiate to macrophages. The cells were then treated with or without GPI for 30 min prior to stimulation with IFN-γ/LPS for 24 hr. Cells in each setting were divided into two parts. One part was used for hydrochloric acid treatment and the ^{13}C-labeled monomer glucose was analysis. Another part is used for ^{13}C-labeled R5P detection by LC/MS/MS. Data are presented as mean ± SEM of n = 3 biologically independent experiments (a, c, f and g) or n = 4 biologically independent experiments (e). *P* values were calculated using one-way ANOVA.

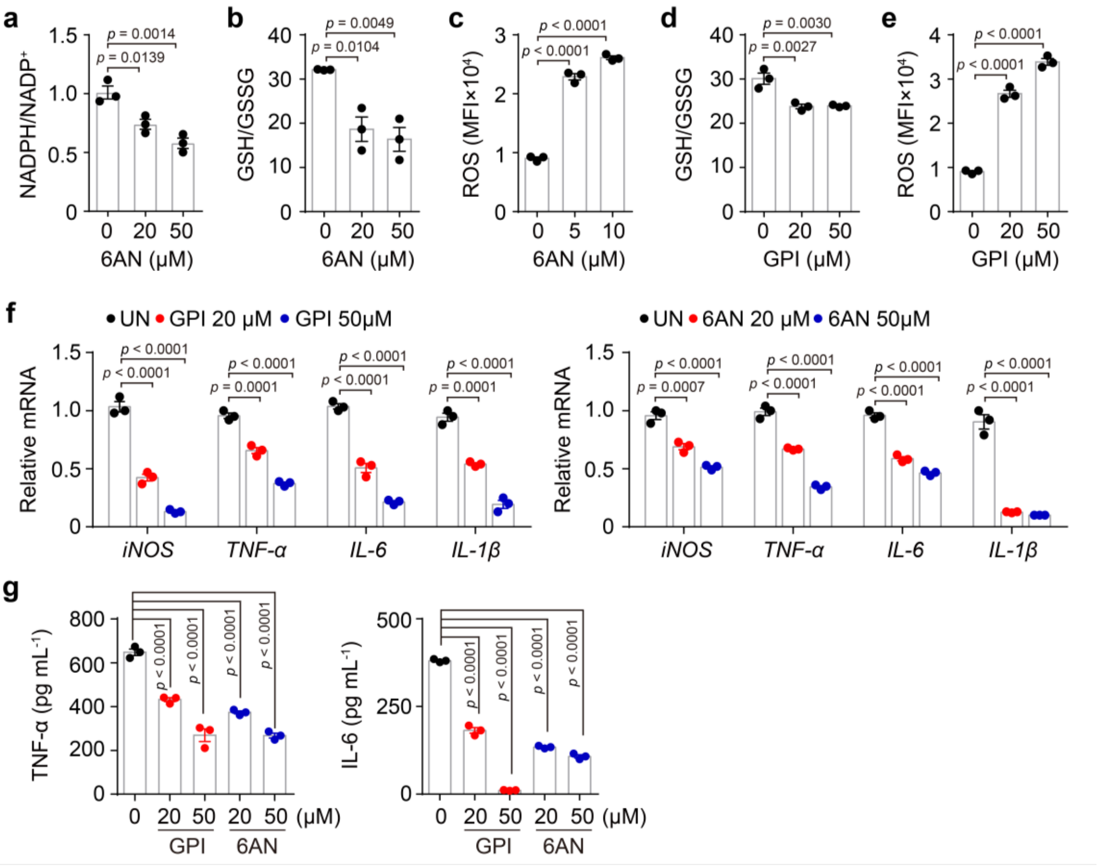

Supplementary Figure 3. The Glycogen-PPP Pathway Regulates the Phenotype, Function and Survival of Inflammatory Macrophages, Related to Fig. 3.

a-e BMDMs were pretreated with 6AN or GPI for 30 min prior to stimulation with IFN-γ/LPS for 24 hr, NADPH/NADP$^+$ (**a**), GSH/GSSG (**b, d**) and ROS (**c, e**) were analyzed. **f, g** BMDMs were pretreated with 6AN or GPI for 30 min prior to stimulation with IFN-γ/LPS for 24 hr, iNOS, TNF-α, IL-6 and *IL-1β* expression was determined by real-time PCR (**f**) and ELISA (**g**). Unless otherwise specified, n = 3 biologically independent experiments were performed. Data are presented as mean ± SEM. *P* values were calculated using one-way ANOVA.

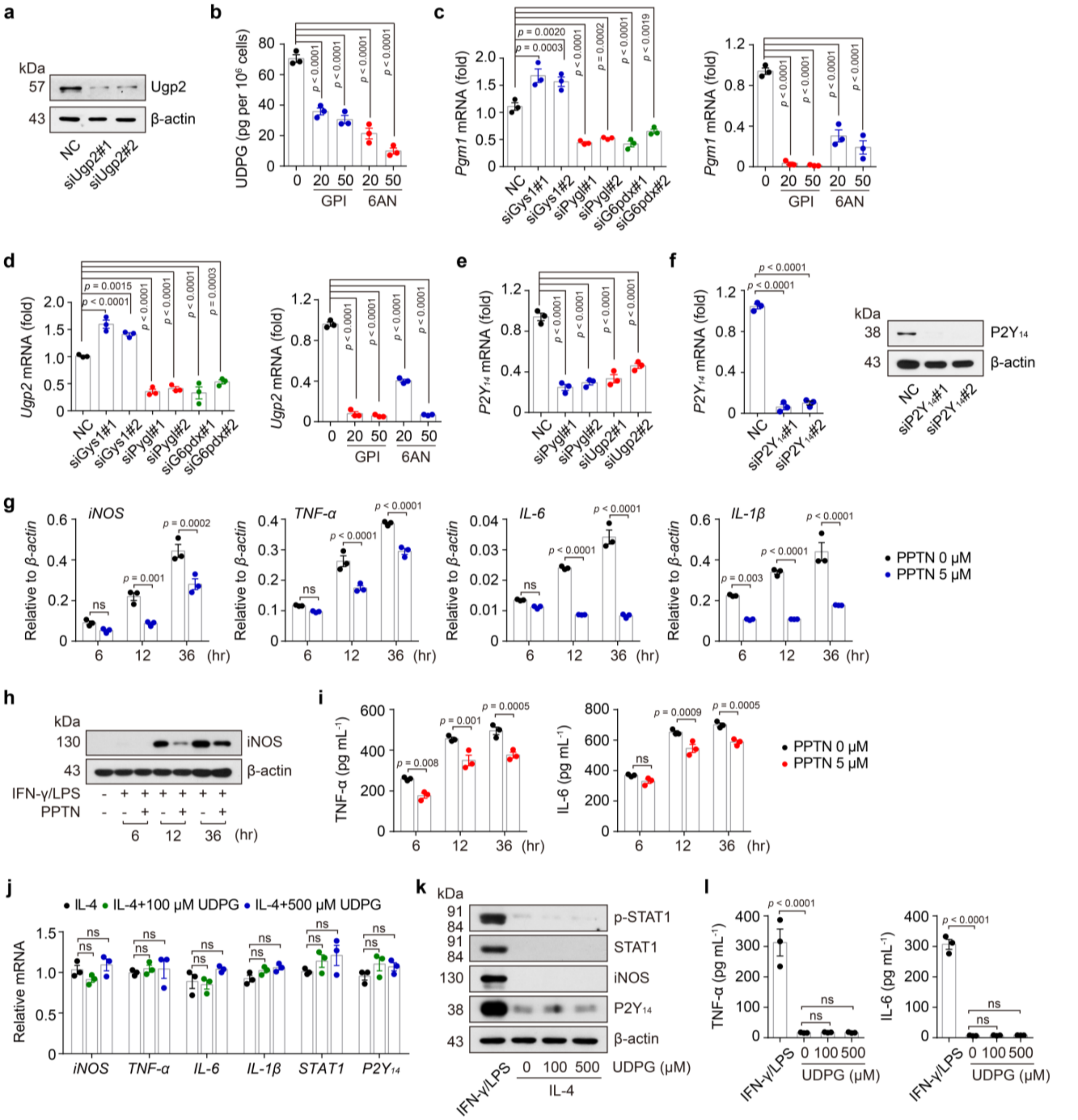

Supplementary Figure 4. Glycogenesis-derived UDPG Regulates Inflammatory Macrophages via $P2Y_{14}$ receptor, Related to Fig. 4.

a BMDMs were pretransfected with Ugp2 siRNA for 24 hr prior to stimulation with IFN-γ/LPS for 36 hr, Ugp2 expression was examined by western blot. **b** BMDMs were pretreated with GPI or 6AN for 30 min prior to stimulation with IFN-γ/LPS for 24 hr, UDPG in supernatants was determined by ELISA. **c, d** BMDMs were pretransfected with Gys1, Pygl or G6pdx siRNA for 24 hr or pretreated with GPI or 6AN for 30 min prior to stimulation with IFN-γ/LPS for 24 hr, Pgm1 and Ugp2 expression was determined by real-time PCR. **e, f** BMDMs were pretransfected with Pygl, Ugp2 or $P2Y_{14}$ siRNA for 24 hr prior to stimulation with IFN-γ/LPS for 24 or 36 hr, $P2Y_{14}$ expression was determined by real-time PCR (**e** and **f**, left) and western blot (**f**, right). **g-i** BMDMs were pretreated with PPTN for 30 min prior to stimulation with IFN-γ/LPS for 6, 12 and 36 hr, iNOS, TNF-α, IL-6 and *IL-1β* expression was determined by real-time PCR (**g**), western blot (**h**) and ELISA (**i**). **j-l** IL-4

stimulated BMDMs were treated with UDPG (0, 100 or 500 μM) for 24 or 36 hr, iNOS, TNF-α, IL-6, *IL-1β*, STAT1 and $P2Y_{14}$ expression was determined by real-time PCR (**j**), western blot (**k**) and ELISA (**l**). IFN-γ/LPS stimulated BMDMs as a positive control. Unless otherwise specified, n = 3 biologically independent experiments were performed. Data are presented as mean ± SEM. *P* values were calculated using one-way ANOVA (b-g, j and l) and two-tailed unpaired Student's *t*-tests (i).

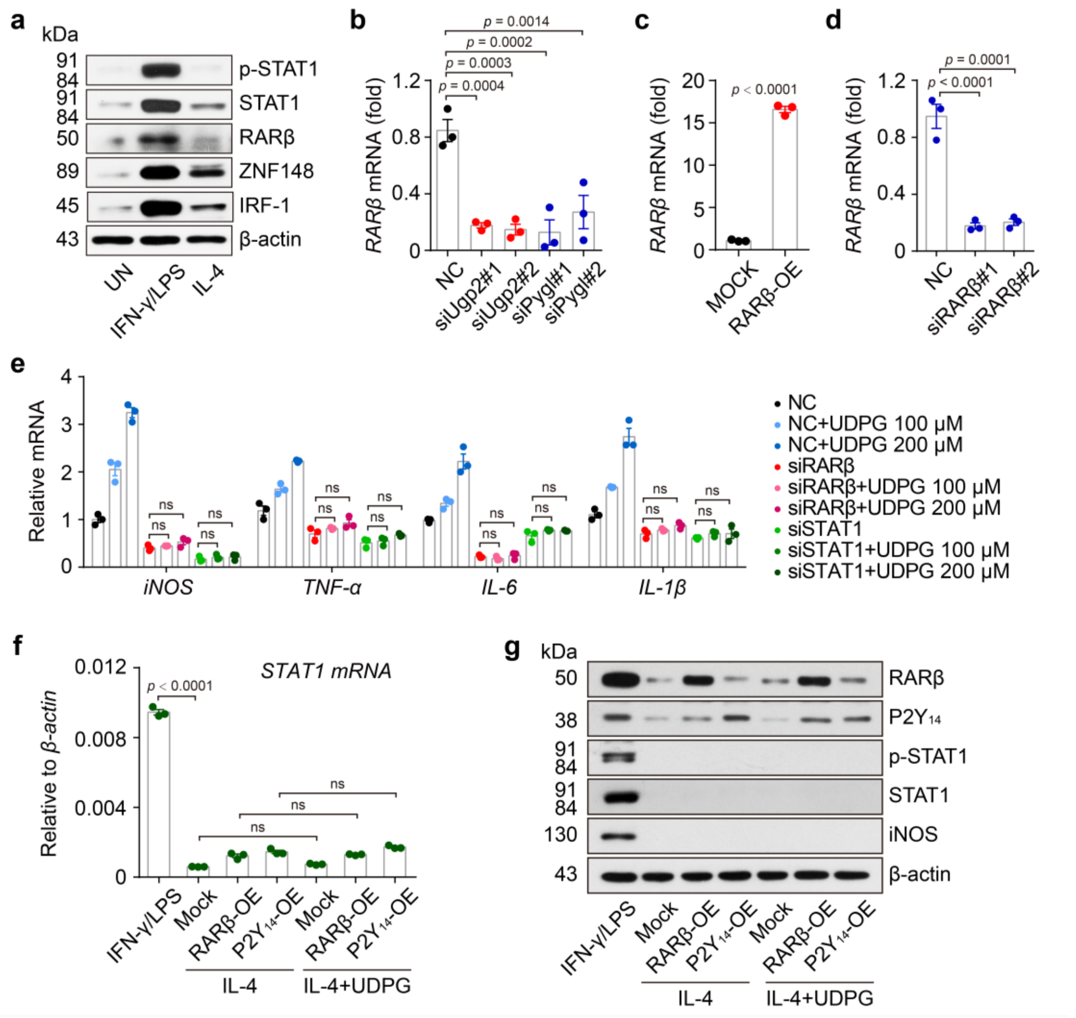

Supplementary Figure 5. UDPG-P2Y$_{14}$ Signaling Regulates STAT1 expression, Related to Fig. 5. **a** STAT1, ZNF-148, IRF-1 and RARβ expression in untreated, IFN-γ/LPS or IL-4 treated BMDMs was analyzed by western blot. **b** BMDMs were pretransfected with Pygl or Ugp2 siRNA for 24 hr prior to stimulation with IFN-γ/LPS for 24 hr, *RARβ* expression was determined by real-time PCR. **c, d** RARβ siRNA or RARβ-overexpression vectors (RARβ-OE) transfected BMDMs were stimulated with IFN-γ/LPS for 24 hr, *RARβ* expression was determined by real-time PCR. **e** RARβ or STAT1 siRNA transfected BMDMs were stimulated with IFN-γ/LPS ± UDPG for 24 hr, *iNOS*, *TNFα*, *IL-6* and *IL-1β* expression was determined by real-time PCR. **f, g** RARβ-OE or P2Y$_{14}$-OE transfected BMDMs were stimulated with IL-4 ± UDPG for 24 or 36 hr, RARβ, STAT1, P2Y$_{14}$ and iNOS expression was determined by real-time PCR (**f**) and western blot (**g**). Unless otherwise specified, n = 3 biologically independent experiments were performed. Data are presented as mean ± SEM. *P* values were calculated using one-way ANOVA (b, d, e and f) and two-tailed unpaired Student's *t*-tests (c).

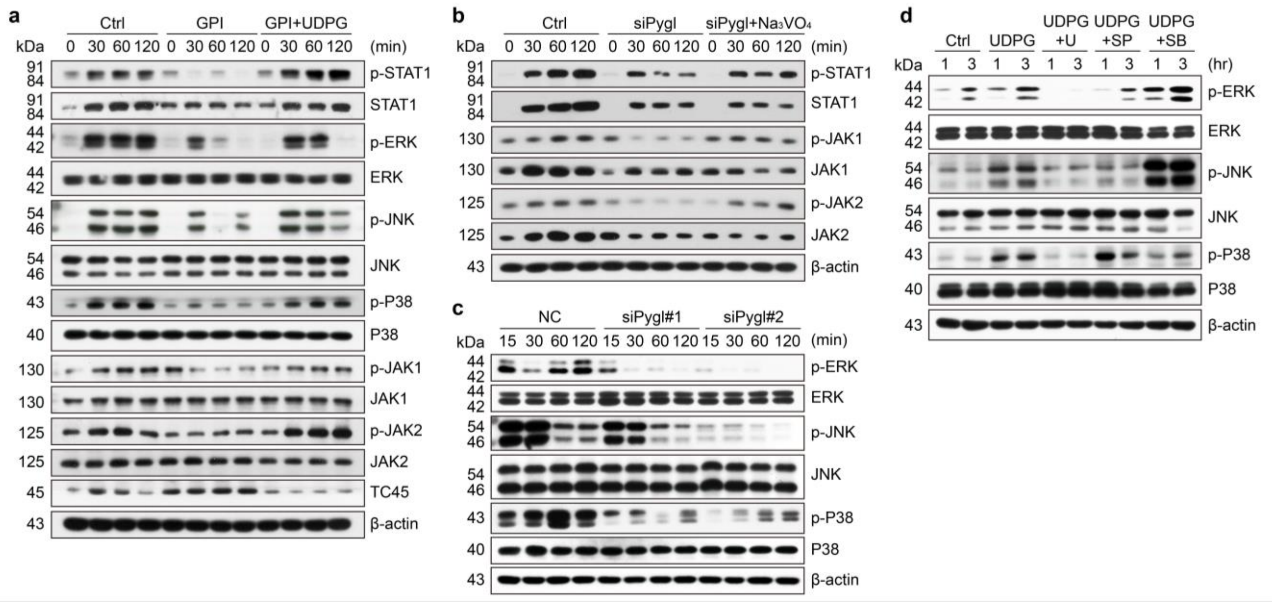

Supplementary Figure 6. UDPG-P2Y14 Signaling Regulates STAT1 Phosphorylation, Related to Fig. 6.

a BMDMs were pretreated with GPI for 30 min prior to stimulation with IFN-γ/LPS ± UDPG, followed by western blot analysis of STAT1, ERK, JNK, P38, JAK1, JAK2 and TC45 from 0 to 120 min after stimulation. **b** BMDMs were pretransfected with Pygl siRNA alone or combined with Na_3VO_4 for 30 min prior to stimulation with IFN-γ/LPS, followed by western blot analysis of STAT1, JAK1 and JAK2 from 0 to 120 min. **c** BMDMs were pretransfected with Pygl siRNA for 24 hr prior to stimulation with IFN-γ/LPS, followed by western blot analysis of ERK, JNK and P38 from 15 to 120 min. **d** IFN-γ/LPS stimulated BMDMs were treated with UDPG alone or combined with U0126, SP600125 or SB203580 for 1 and 3 hr, followed by western blot analysis of ERK, JNK and P38. Data are from one experiment representative of three experiments.

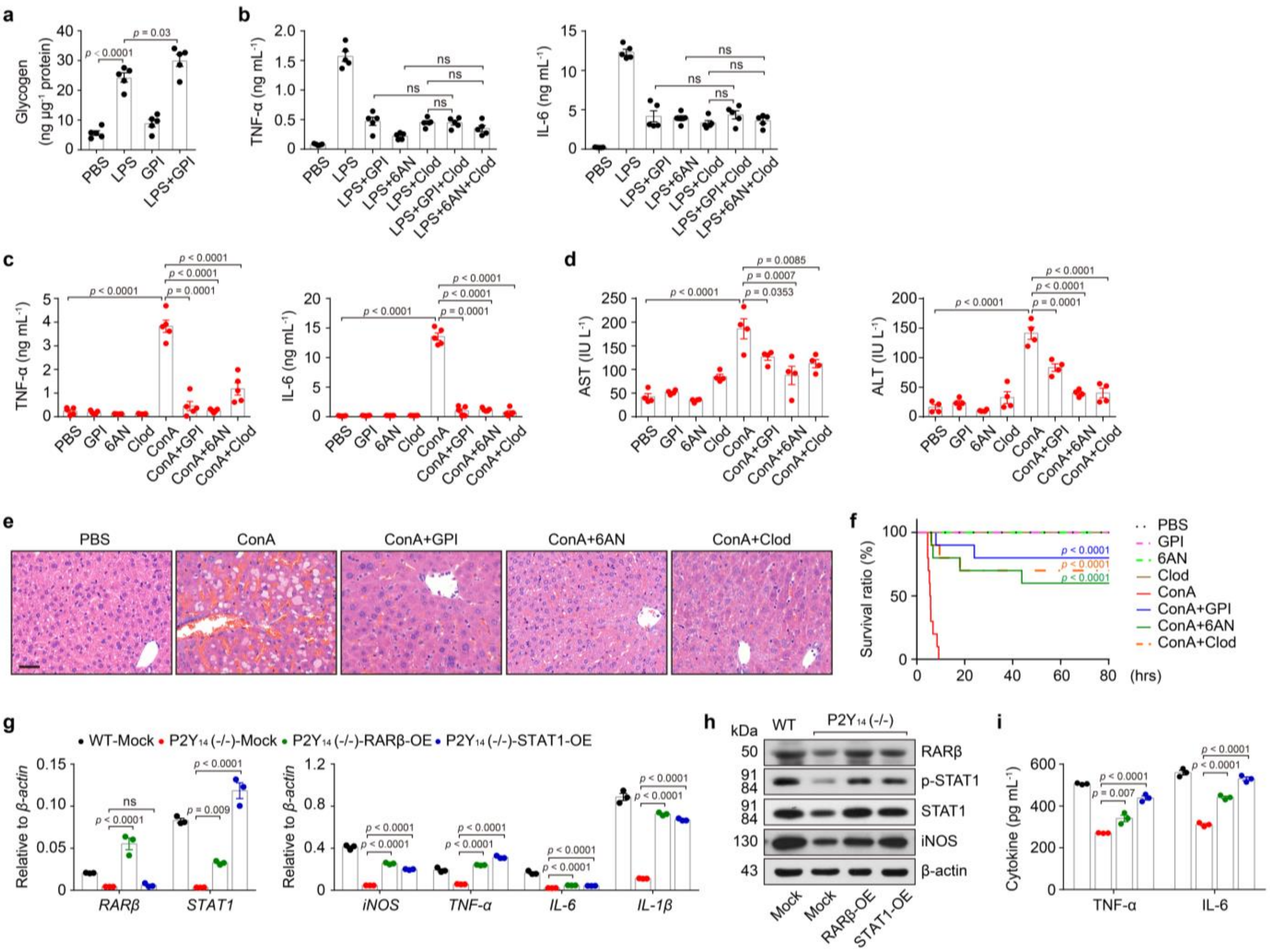

Supplementary Figure 7. Glycogen Metabolism Regulates Macrophage-induced Inflammatory Responses *in vivo,* Related to Fig. 7.

a C57BL/6J mice were treated with GPI (10 µg g^{-1}, i.p., 12 and 4 hr before LPS injection) followed by i.p. injection of 20 µg g^{-1} body weight LPS. Four hours later, glycogen levels in peritoneal macrophages were detected by colorimetric assay (n = 5 mice per group). **b** C57BL/6J mice were treated with GPI (10 µg g^{-1}, i.p., 12 and 4 hr before LPS injection), 6AN (15 µg g^{-1}, i.p., 12 and 4 hr before LPS injection), clodronate liposomes (Clod, 10 µL g^{-1}, i.p., 36 and 4 hr before- and 36 hr after- LPS injection), GPI+Clod or 6AN+Clod, followed by i.p. injection of 20 µg g^{-1} body weight LPS. Four hours later, serum levels of TNF -α and IL-6 were detected by ELISA, n = 5 mice per group. **c-f** C57BL/6J mice were treated with GPI, 6AN or clodronate liposomes, followed by i.v. injection of 15 µg g^{-1} body weight ConA. Four hours later, serum levels of TNF-α and IL-6, n = 5 mice per group (**c**), transaminase AST and ALT, n = 4 mice per group (**d**) were detected by ELISA. Liver sections were stained with H&E, scale bar, 40 µm (**e**). The long-term survival of ConA induced hepatitis was recorded, n = 10 mice per group, *p* values are presented relative to ConA group (**f**). **g-i** RARβ-OE or STAT1-OE transfected BMDMs from WT or $P2Y_{14}$ (-/-) mice were stimulated with IFN-γ/LPS for 24 or 36 hr, RARβ, STAT1, iNOS, TNF-α, IL-6 and *IL-1β* expression was determined by real-time PCR, n = 3 mice per group (**g**) western blot (**h**) and ELISA, n = 3 mice per group (**i**). Unless otherwise specified, n = 3 independent experiments were performed. Data are presented as mean ± SEM. *P* values were calculated using one-way ANOVA (a-d, g and i) and two-sided log-rank (Mantel-Cox) test (f).

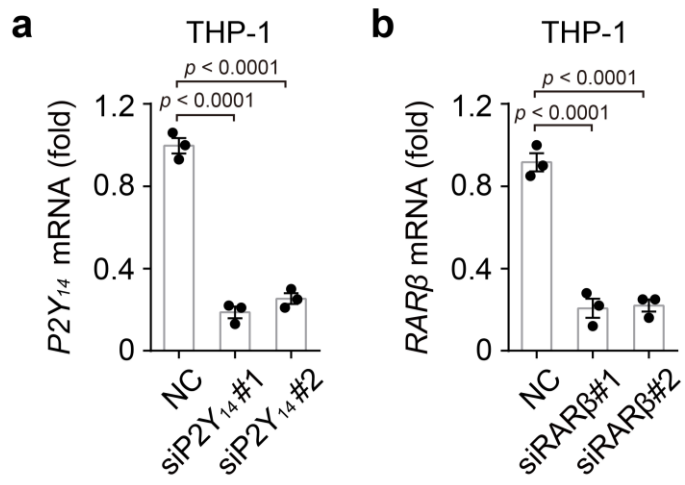

Supplementary Figure 8. Glycogen Metabolism Regulates Inflammatory Responses in Human Macrophages and Septic Patients, Related to Fig. 8.

a $P2Y_{14}$ siRNA transfected THP-1 cells were stimulated with IFN-γ/LPS for 24 hr, $P2Y_{14}$ expression was determined by real-time PCR. **b** RARβ siRNA transfected THP-1 cells were stimulated with IFN-γ/LPS for 24 hr, *RARβ* expression was determined by real-time PCR. Data are presented as mean ± SEM of n = 3 biologically independent experiments. *P* values were calculated using one-way ANOVA.

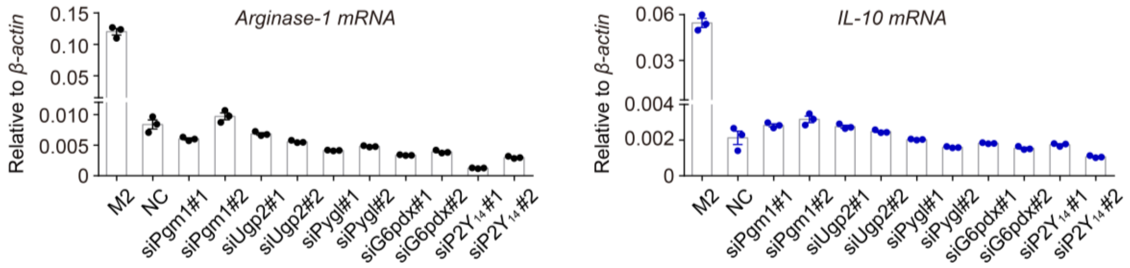

Supplementary Figure 9. Blocking the glycogen-PPP or UDPG-P2Y14 pathway did not switch inflammatory macrophages toward anti-inflammatory phenotype. BMDMs were pretransfected with siRNAs (Pgm1, Ugp2, Pygl, G6pdx or $P2Y_{14}$) for 24 hr prior to stimulation with IFN-γ/LPS for 24 hr, *arginase-1* and *IL-10* expression was determined by real-time PCR. Data are presented as mean ± SEM of n = 3 biologically independent experiments.

Supplementary Table 1. Clinical information of Sepsis and SIRS patients.

1. Sepsis patients

NO	Age	Gender	WBC ($\times 10^9$ L^{-1})	Sequential Organ Failure Assessment, SOFA					
				Respiration	Coagulation	Liver	Cardiovascular	Nervous	Renal
				PaO_2 mm Hg	Platelets ($\times 10^3$ μL^{-1})	Bilirubin (μmol L^{-1})	Dopamine mg (kg·min)$^{-1}$	Glasgow score	Creatinine (μmol L^{-1})
1	55	M	12.69	321	83	27.0	0	15（4+5+6）	116.8
2	58	M	21.63	212	75	29.8	6.0	8（2+2+4）	76.6
3	44	M	13.33	280	97	31.2	0	12（3+4+5）	85.2
4	49	F	14.14	243	78	41.6	0	12（3+4+5）	261.2
5	60	F	13.83	332	50	60.0	0	14（3+5+6）	62.0
6	66	M	18.27	355	78	26.3	5.0	13（3+4+6）	82.3
7	33	M	23.08	86	84	32.6	8.0	2（1+T+1）	106.6
8	58	M	2.52	244	30	187.3	7.0	10（2+4+4）	158.0
9	54	M	14.30	282	79	89.6	0	14（3+5+6）	139.0
10	52	M	18.31	366	250	30.7	3.0	13（3+4+6）	217.4
11	50	M	12.60	265	94	16.9	0	14（3+5+6）	129.2
12	37	M	14.60	347	73	14.1	0	14（3+5+6）	278.1
13	35	F	12.37	351	268	284.6	0	13（3+4+6）	246.2
14	65	M	14.83	189	172	51.3	0	12（3+4+5）	102.6
15	62	F	13.62	292	172	63.6	0	12（3+4+5）	83.8
16	65	M	22.50	118	76	27.4	3.5	6（2+T+6）	132.8
17	69	M	13.00	476	94	12.2	0	15（4+5+6）	245.6
18	50	M	19.90	355	220	14.9	4.0	13（3+4+6）	270.6
19	45	F	10.87	352	57	39.7	0	13（3+4+6）	506.7
20	65	M	17.70	260	75	8.8	2.0	13（3+4+6）	98.8
21	55	M	14.09	440	13	841.1	0	14（3+5+6）	54.3
22	71	M	12.48	284	75	21.4	0	14（3+5+6）	101.6
23	69	F	2.58	136	40	27.3	7.0	7（3+T+4）	43.5
24	48	M	29.77	277	203	22.5	5.5	12（3+4+5）	251.9
25	44	M	16.60	292	83	95.7	0	11（2+4+5）	140.0

2. SIRS patients

NO	Age	Gender	WBC ($\times10^9$ L^{-1})	Sequential Organ Failure Assessment, SOFA					
				Respiration	Coagulation	Liver	Cardiovascular	Nervous	Renal
				PaO_2 mm Hg	Platelets ($\times10^3$ μL^{-1})	Bilirubin (μmol L^{-1})	Dopamine mg $(kg\cdot min)^{-1}$	Glasgow score	Creatinine (μmol L^{-1})
1	3	M	17.85	352	274	16.6	0	15（4+5+6）	32.9
2	6	M	12.32	321	150	11.3	0	15（4+5+6）	61.2
3	56	F	13.47	317	146	18.1	0	15（4+5+6）	107.7
4	49	M	13.54	325	229	18.6	0	15（4+5+6）	62.4
5	55	F	12.56	308	123	21.8	0	15（4+5+6）	54.2
6	58	F	13.05	330	130	25.3	0	15（4+5+6）	36.8
7	54	M	14.31	301	307	18.6	0	15（4+5+6）	61.3
8	54	F	17.91	366	176	7.1	0	15（4+5+6）	48.6
9	4	M	15.78	402	173	14.0	0	15（4+5+6）	32.8
10	46	M	16.76	310	139	11.1	0	15（4+5+6）	65.1
11	3	M	12.15	398	303	9.6	0	15（4+5+6）	35.3
12	65	F	12.45	300	149	5.7	0	15（4+5+6）	76.9
13	42	M	19.50	371	440	6.0	0	15（4+5+6）	99.8
14	3	F	16.11	389	376	20.2	0	15（4+5+6）	42.7
15	51	M	18.04	311	133	27.2	0	15（4+5+6）	91.9
16	5	F	13.03	368	257	7.3	0	15（4+5+6）	31.1
17	37	M	16.17	325	298	14.8	0	15（4+5+6）	104.3
18	4	F	12.68	422	232	7.0	0	15（4+5+6）	58.5
19	56	M	16.79	309	430	10.1	0	15（4+5+6）	39.6
20	3	F	12.25	386	330	26.0	0	15（4+5+6）	31.7
21	59	M	14.56	362	202	12.8	0	15（4+5+6）	129.1
22	55	M	18.55	345	273	15.7	0	15（4+5+6）	82.4
23	64	M	19.31	301	251	11.5	0	15（4+5+6）	134.4
24	60	M	16.00	301	158	19.2	0	15（4+5+6）	57.7
25	59	F	12.35	335	232	16.4	0	15（4+5+6）	92.0
26	38	M	14.70	382	257	25.6	0	15（4+5+6）	98.1
27	65	F	19.46	341	137	18.3	0	15（4+5+6）	79.2
28	50	M	19.02	327	322	21.0	0	15（4+5+6）	34.7

Supplementary Table 2. siRNA Sequence.

Gene	siRNA	Sequence
Mouse HK1	siRNA#1	GCTGCTGAATAAAGCCATT
	siRNA#2	GATCGAGAGTGACCGATTA
Mouse HK2	siRNA#1	CCAAAGATGTCTCGGATAT
	siRNA#2	GAAGGATGAAGGTGGAAAT
Mouse HK3	siRNA#1	GTCTGAAGCTTGGGTTCAA
	siRNA#2	ACTGCATCGTGGACTTCCA
Mouse Pygl	siRNA#1	CCATTTACCAGCTTGGATT
	siRNA#2	CCAATCAGCCAGACCTCTT
Mouse $P2Y_{14}$ receptor	siRNA#1	GCCGCAATATCTTCAGCAT
	siRNA#2	GCATGGAGCTCAAAAACGA
Mouse Ugp2	siRNA#1	GCAAAGGATGTGTCTTATT
	siRNA#2	CCACAGTGGATCTTTATAT
Mouse Stat1	siRNA#1	CTGTGATGTTAGATAAACA
	siRNA#2	GCAGCACAACATACGGAAA
Mouse Gys1	siRNA#1	CCTGGAGAATTTCAATGTA
	siRNA#2	GCACCTGGACTTC AACCTA
Mouse G6pdx	siRNA#1	CCTCAACAGCCACATGAAT
	siRNA#2	CCAACAGTGCAAGCGTAAT
Mouse RARβ	siRNA#1	GCTGGAGAATTCTGAAGGA
	siRNA#2	GAACGTGTAATTACCTTGA
Mouse Pgm1	siRNA#1	GTTCCTGTATACCTCTTCT
	siRNA#2	CCATGATCAGGGCACAATT
Mouse TC45	siRNA#1	ACAGAGTGATGGTTGAGAA
	siRNA#2	AGAGAATAGGTTCAGAAGA
Human $P2Y_{14}$ receptor	siRNA#1	CCTACTCAATGGAGTGTCA
	siRNA#2	GAGTGTTAGGGAGGTTACA
Human RARβ	siRNA#1	AGACGGCCTTACCCTAAAT
	siRNA#2	GATCGTGGAGTTTGCTAAA

Supplementary Table 3. Primer sequence.

Gene	Primer	Primer sequence
Mouse *Gys1*	FW	CACAGAACGGTTGTCGGACTTG
	RV	AGGTGAAGTGGTCTGGAAAGGC
Mouse *Pgm1*	FW	AGCCAATGACCCAGATGCTGAC
	RV	TCCAGGAAGTGAAGAGCCACCA
Mouse *Ugp2*	FW	CTGATGAACCCACCCAATGGGA
	RV	GAGCGATTTCCACCAGTCTCAG
Mouse *Pck1*	FW	GGCGATGACATTGCCTGGATGA
	RV	TGTCTTCACTGAGGTGCCAGGA
Mouse *Fbp1*	FW	TGCTGAAGTCGTCCTACGCTAC
	RV	TTCCGATGGACACAAGGCAGTC
Mouse *G6pase*	FW	AGGTCGTGGCTGGAGTCTTGTC
	RV	GTAGCAGGTAGAATCCAAGCGC
Mouse *HK1*	FW	CGGAATGGGGAGCCTTTGG
	RV	GCCTTCCTTATCCGTTTCAATGG
Mouse *HK2*	FW	ATGCGTAATGTGGAACTGGTG
	RV	GCTGATCATCTTCTCAAACCTCTG
Mouse *HK3*	FW	TGCTGCCCACATACGTGAG
	RV	GCCTGTCAGTGTTACCCACAA
Mouse *Slc2a1*	FW	CAGTTCGGCTATAACACTGGTG
	RV	GCCCCCGACAGAGAAGATG
Mouse *Slc2a2*	FW	TCAGAAGACAAGATCACCGGA
	RV	GCTGGTGTGACTGTAAGTGGG
Mouse *Pygl*	FW	GGCAGAAGTGGTGAACAATGACC
	RV	TCCGATAGGTCTGTGGCTGGAA
Mouse *Pygm*	FW	ATGGCACACCTGTGCATTGCTG
	RV	CGAGGAGTGATGCCATTGGTCT
Mouse *G6pdx*	FW	GACCAAGAAGCCTGGCATGTTC
	RV	AGACATCCAGGATGAGGCGTTC
Mouse *6Pgd*	FW	CATCGCTGCAAAAGTGGGAACC
	RV	AGCCTCACAGATGAGCTGCATG
Mouse *iNOS*	FW	GAGACAGGGAAGTCTGAAGCAC
	RV	CCAGCAGTAGTTGCTCCTCTTC
Mouse *TNF-α*	FW	GGTGCCTATGTCTCAGCCTCTT
	RV	GCCATAGAACTGATGAGAGGGAG
Mouse *IL-6*	FW	TAGTCCTTCCTACCCCAATTTCC
	RV	TTGGTCCTTAGCCACTCCTTC

Mouse *IL-1β*	FW	GCAACTGTTCCTGAACTCAACT
	RV	ATCTTTTGGGGTCCGTCAACT
Mouse *P2Y*$_{14}$	FW	ACCTCCGTCAAGAGGAAGTCCA
	RV	GCTGTAGTGACCTTCCGTCTGA
Mouse *Stat1*	FW	GCCTCTCATTGTCACCGAAGAAC
	RV	TGGCTGACGTTGGAGATCACCA
Mouse *RARβ*	FW	GCTTCGTTTGCCAGGACAAGTC
	RV	TGGCATCGGTTCCTAGTGACCT
Mouse *β-actin*	FW	GGCTGTATTCCCCTCCATCG
	RV	CCAGTTGGTAACAATGCCATGT
Human *Gys1*	FW	CCGCTATGAGTTCTCCAACAAGG
	RV	AGAAGGCAACCACTGTCTGCTC
Human *Pgm1*	FW	TGATGGACGCGAGCAAACTGTC
	RV	ATGTCCTCCACACTCTGCTTGC
Human *Ugp2*	FW	GCAGGAGCAAAATGCCATTGACA
	RV	CAGAAAACGGCTCCTTGGCACA
Human *Pygl*	FW	CACTTCAGTGGCAGATGTGGTG
	RV	GCAGTGGAAATCTGCTCTGACAG
Human *Pygm*	FW	ATGGCACACCTGTGCATTGCTG
	RV	CGAGGAGTGATGCCATTGGTCT
Human *iNOS*	FW	GCTCTACACCTCCAATGTGACC
	RV	CTGCCGAGATTTGAGCCTCATG
Human *TNF-α*	FW	CTCTTCTGCCTGCTGCACTTTG
	RV	ATGGGCTACAGGCTTGTCACTC
Human *IL-6*	FW	ACTCACCTCTTCAGAACGAATTG
	RV	CCATCTTTGGAAGGTTCAGGTTG
Human *IL-1β*	FW	CCACAGACCTTCCAGGAGAATG
	RV	GTGCAGTTCAGTGATCGTACAGG
Human *P2Y*$_{14}$	FW	GCCGCAACATATTCAGCATCGTG
	RV	GCTGTAATGAGCTTCGGTCTGAC
Human *Stat1*	FW	ATGGCAGTCTGGCGGCTGAATT
	RV	CCAAACCAGGCTGGCACAATTG
Human *RARβ*	FW	GGTTTCACTGGCTTGACCATCG
	RV	CCGTCTGAGAAAGTCATGGTGTC
Human *β-actin*	FW	CACCATTGGCAATGAGCGGTTC
	RV	AGGTCTTTGCGGATGTCCACGT

Full unedited and uncropped gels

Figure 1e

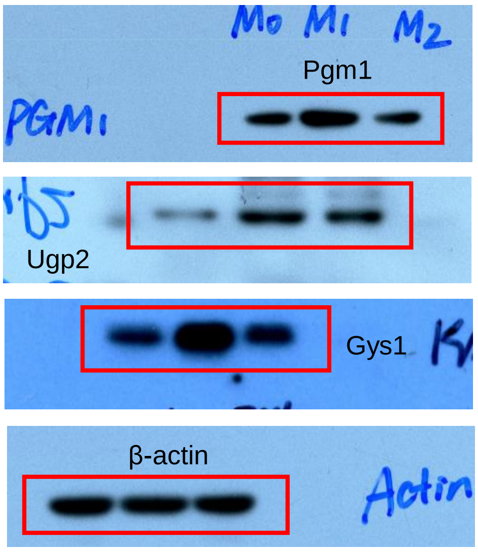

Figure 1k

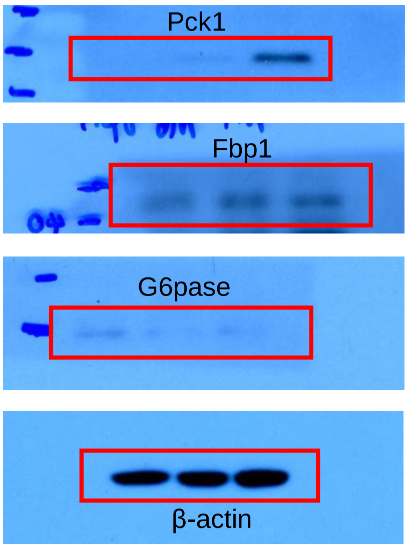

Figure 2b

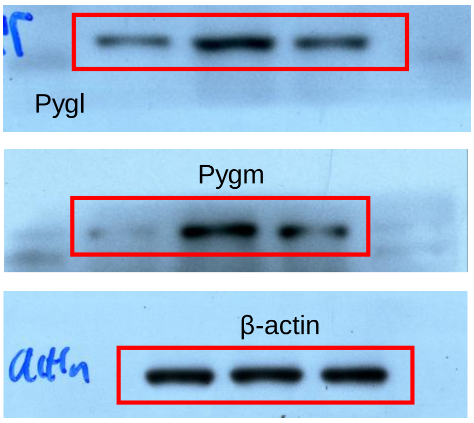

Figure 2f

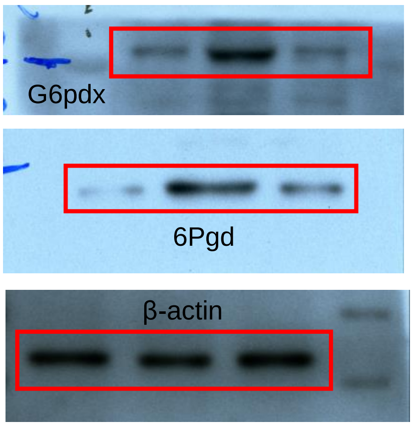

Figure 3i

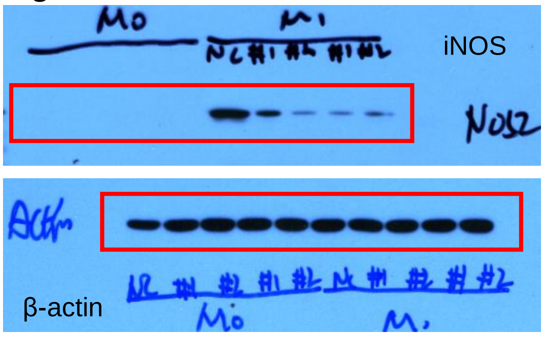

Figure 4e

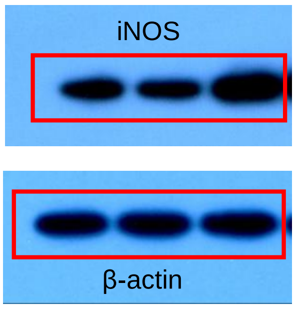

Figure 4f

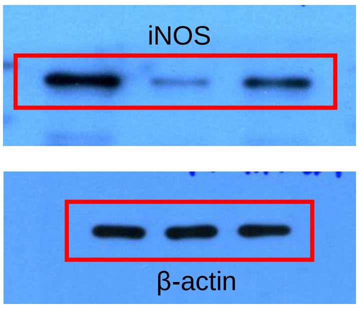

Figure 4g

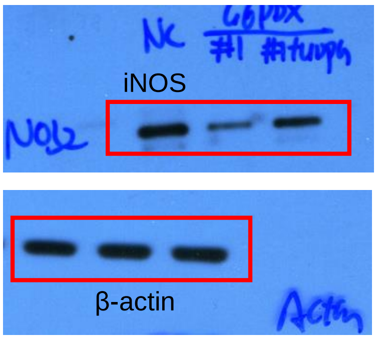

Figure 4k

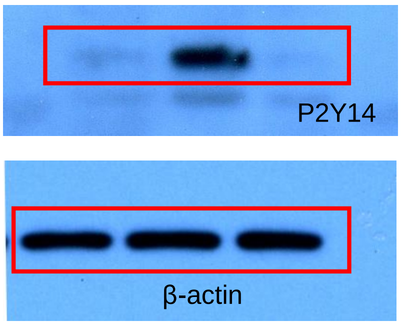

Figure 4m

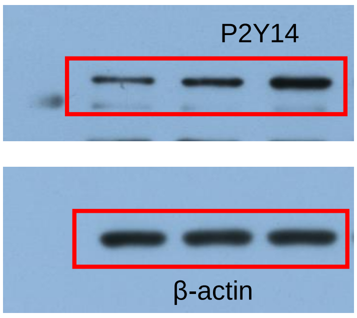

Figure 4o

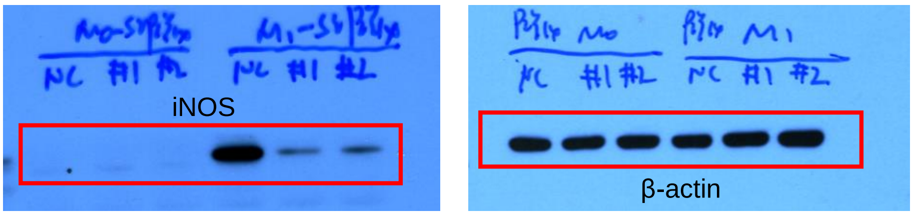

Figure 5c

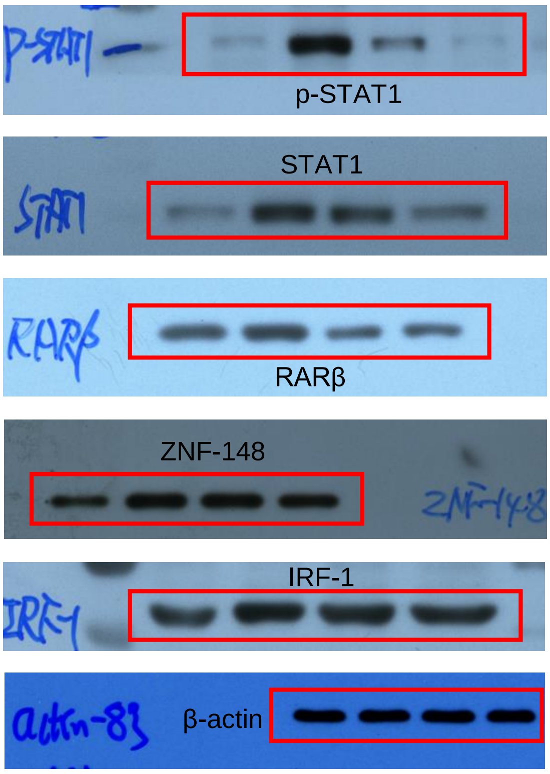

Figure 5d

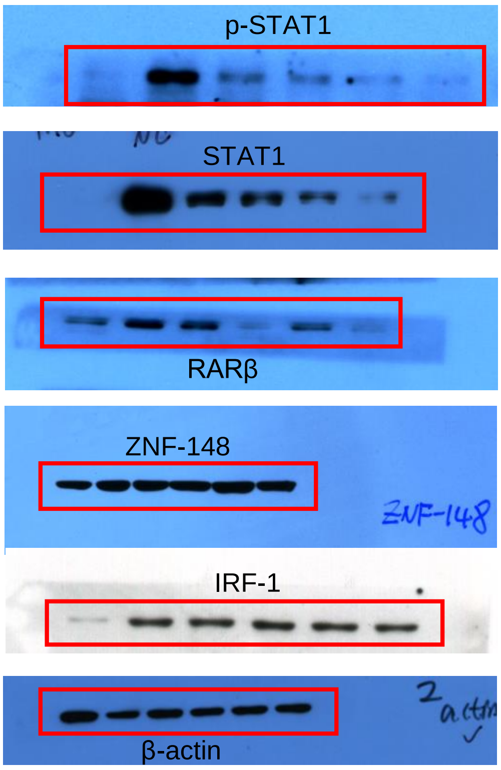

Figure 5k

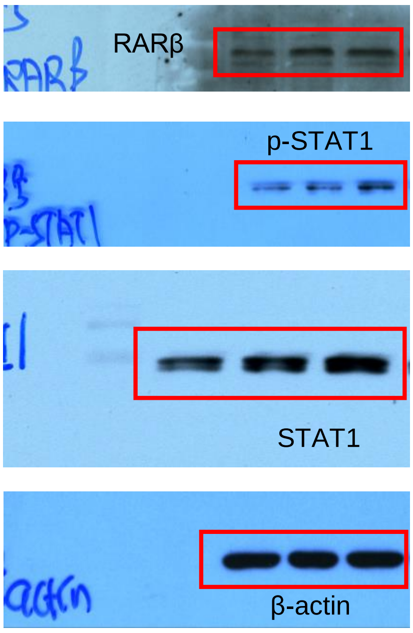

Figure 5m

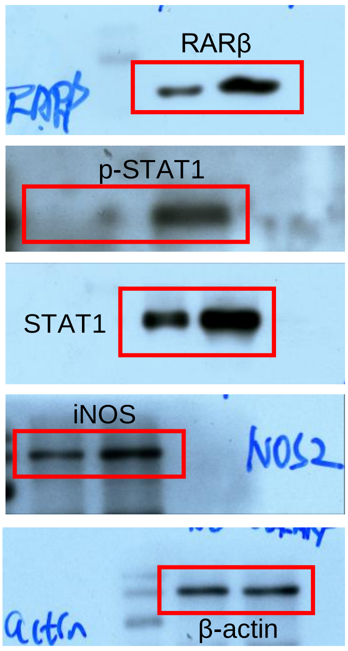

Figure 5p

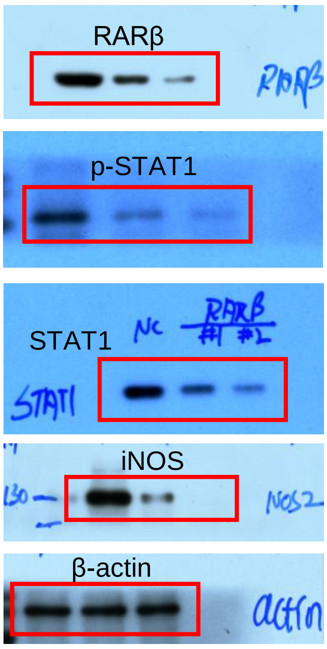

Figure 6a

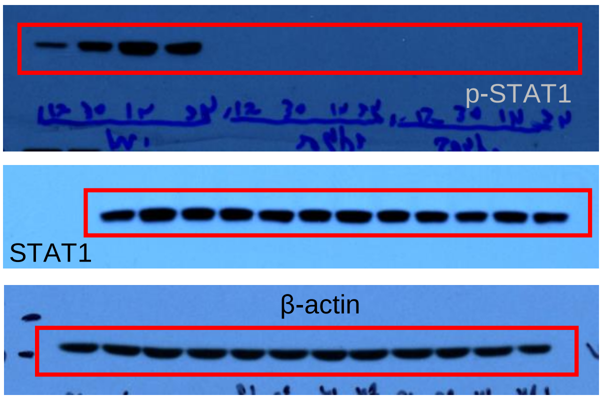

Figure 6b

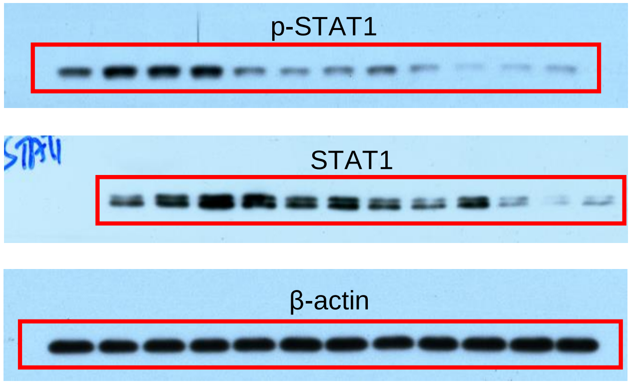

Figure 6d

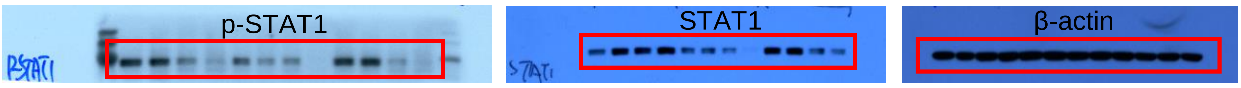

Figure 6e

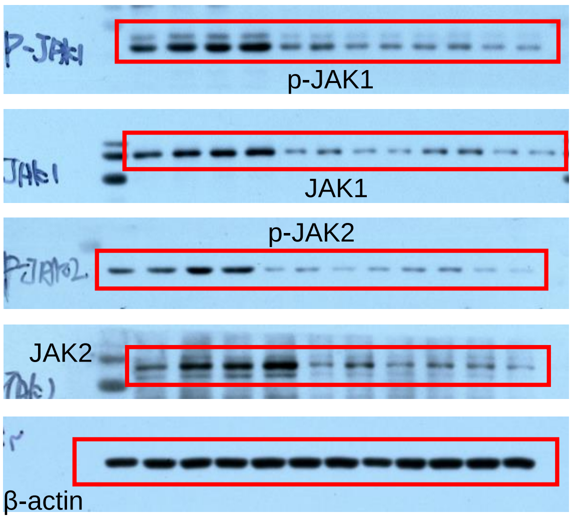

Figure 6f

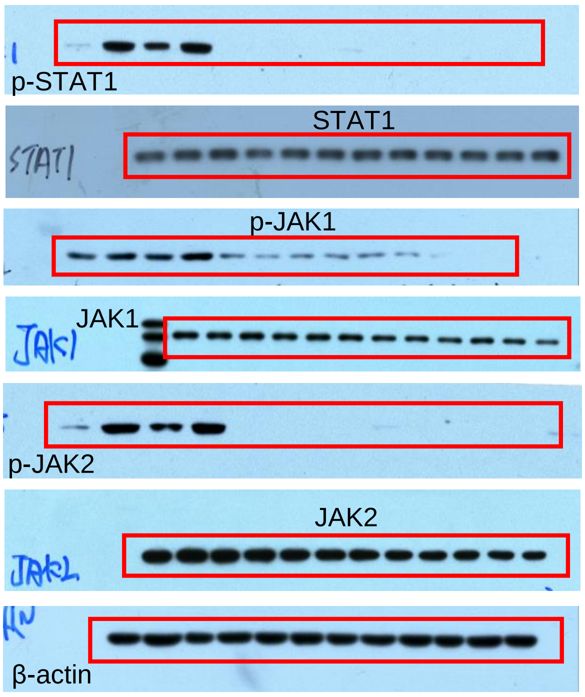

Figure 6g

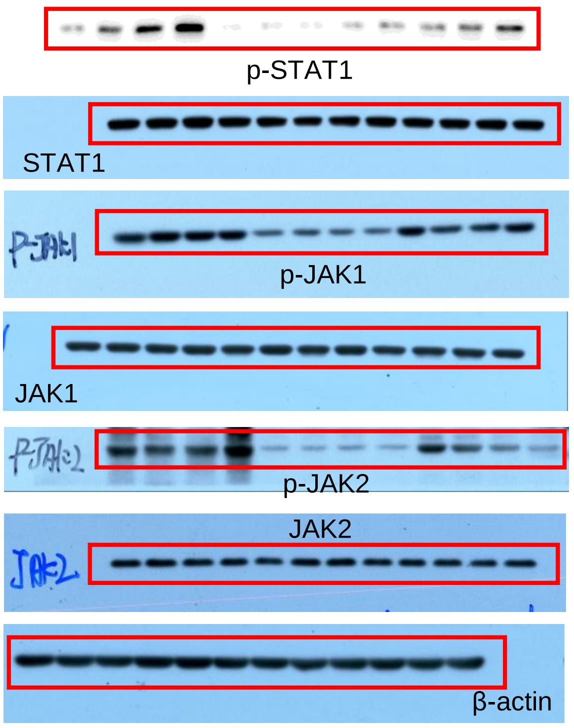

Figure 6h

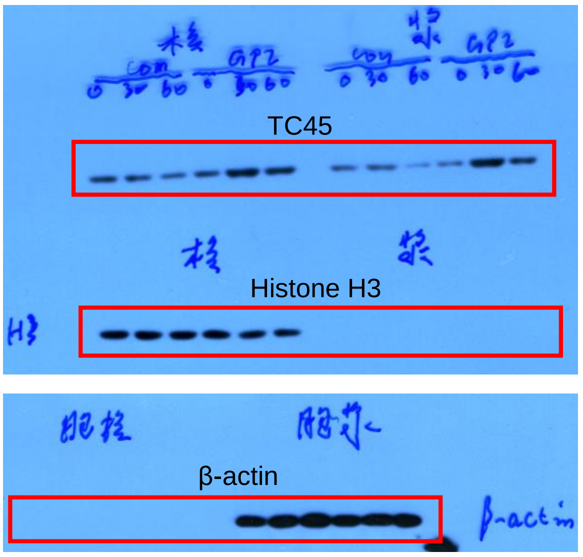

Figure 6i

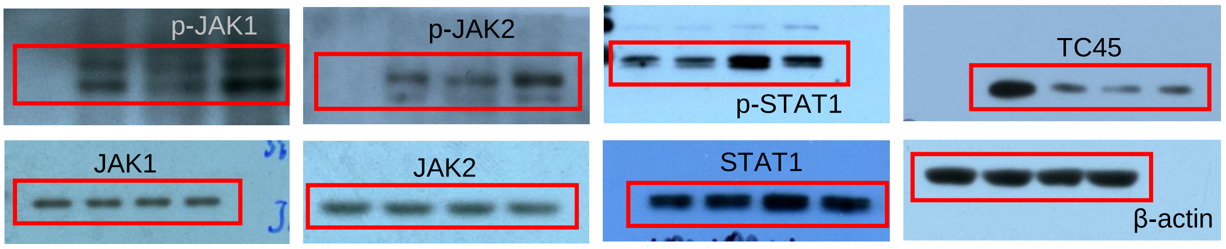

Figure 6j

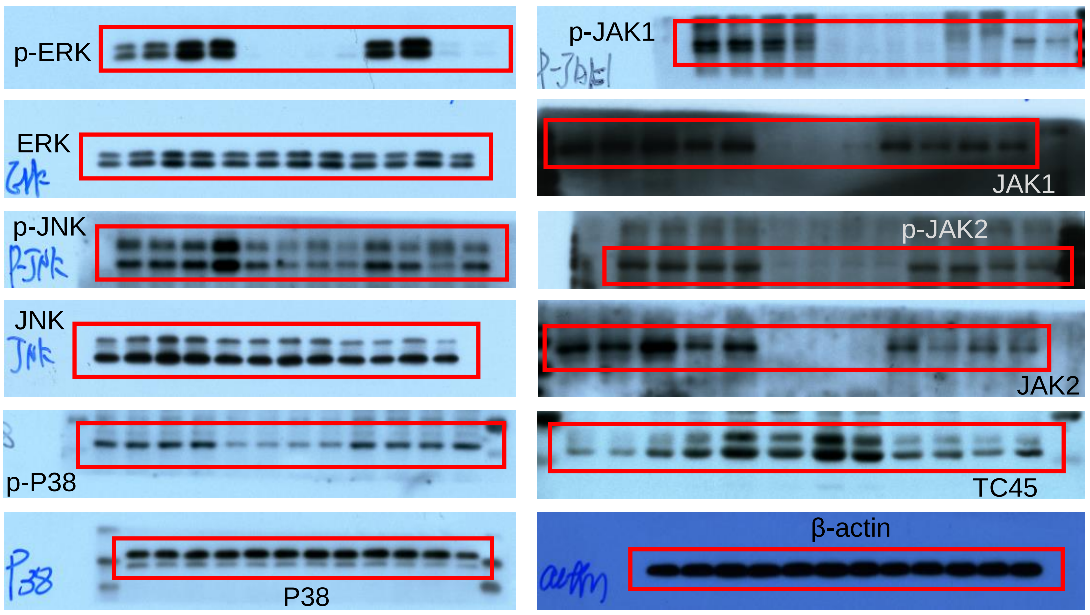

Figure 6k

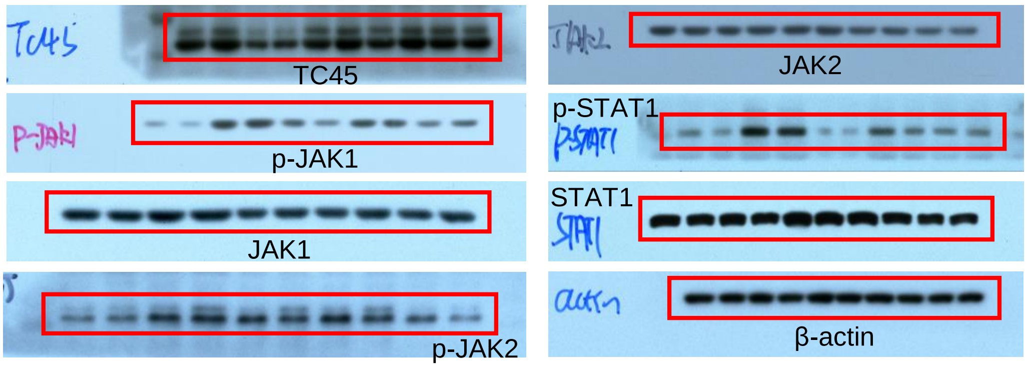

Figure 7n

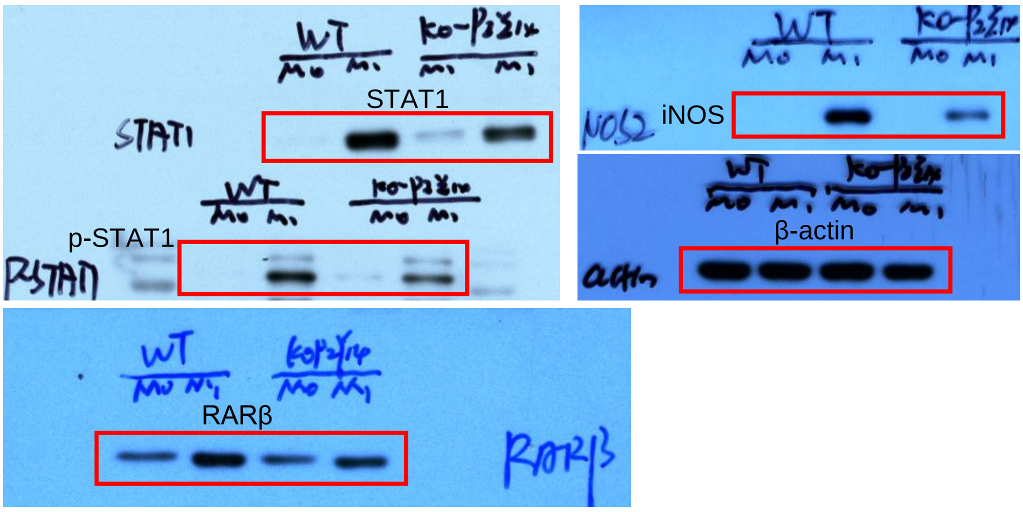

Figure 8e

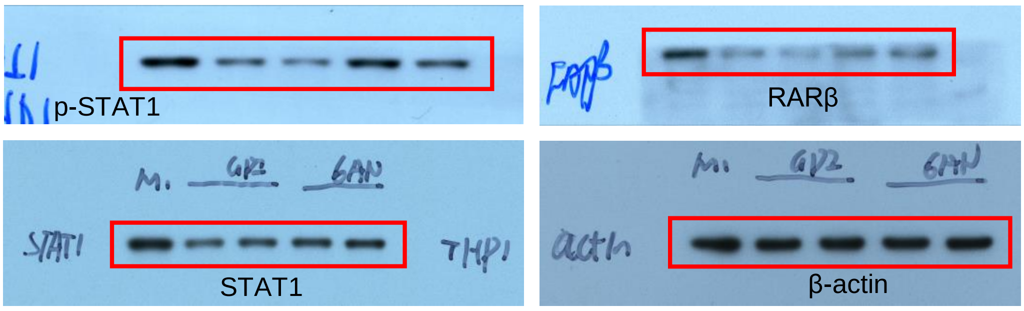

Figure 8g

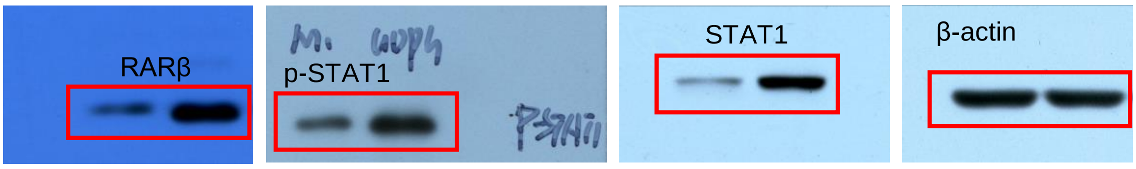

Figure S1c

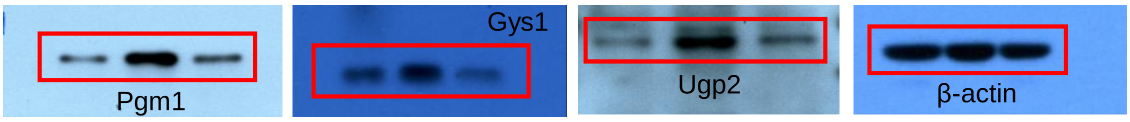

Figure S1f

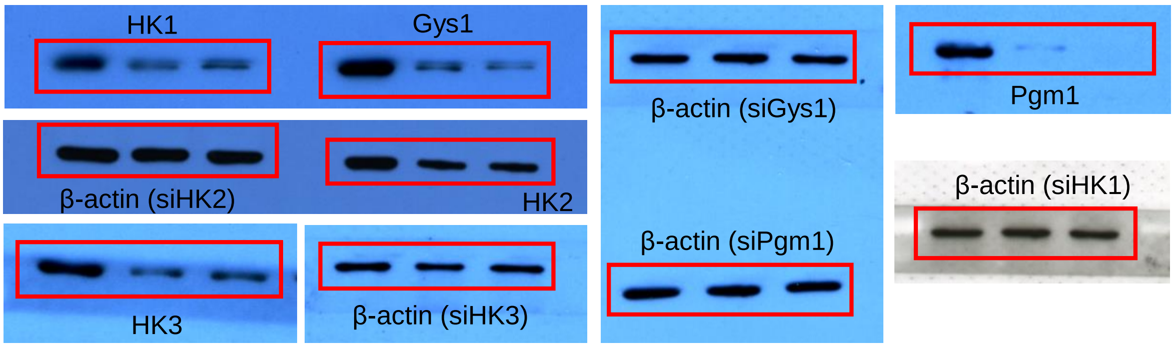

Figure S2b

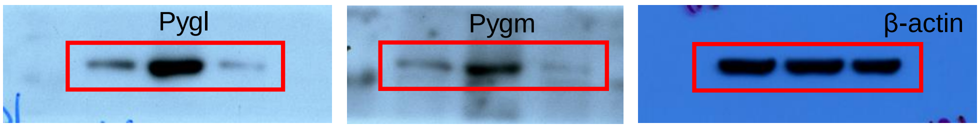

Figure S2d

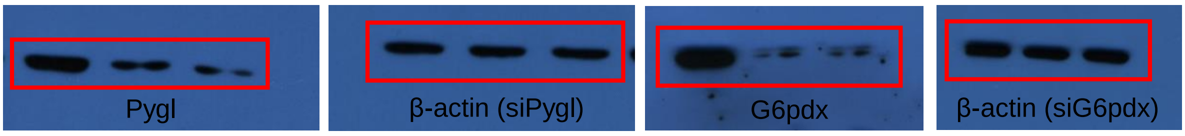

Figure S4a

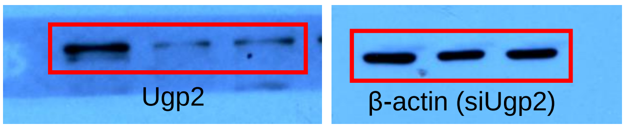

Figure S4f

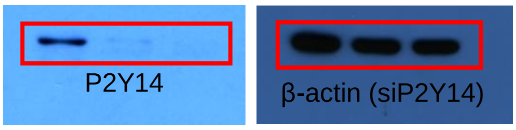

Figure S4h

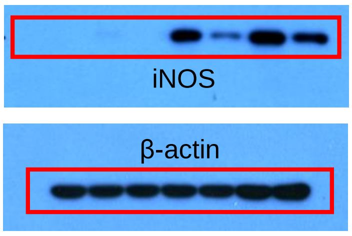

Figure S4k

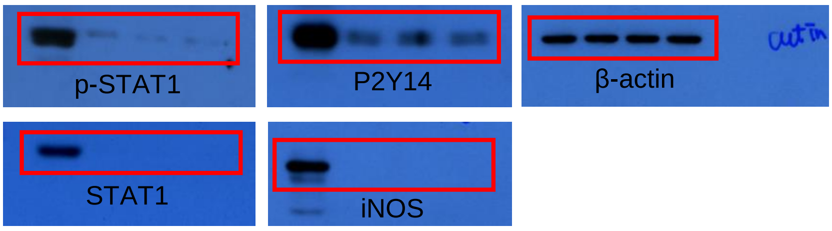

Figure S5a

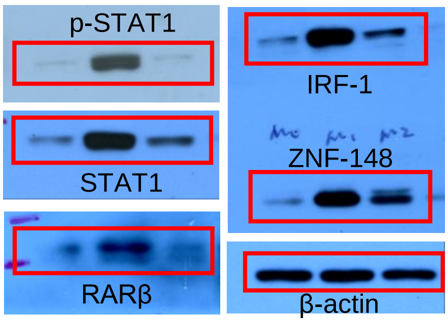

Figure S5g

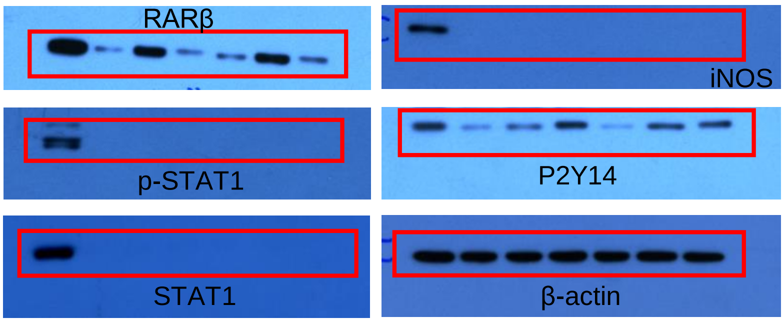

Figure S6a

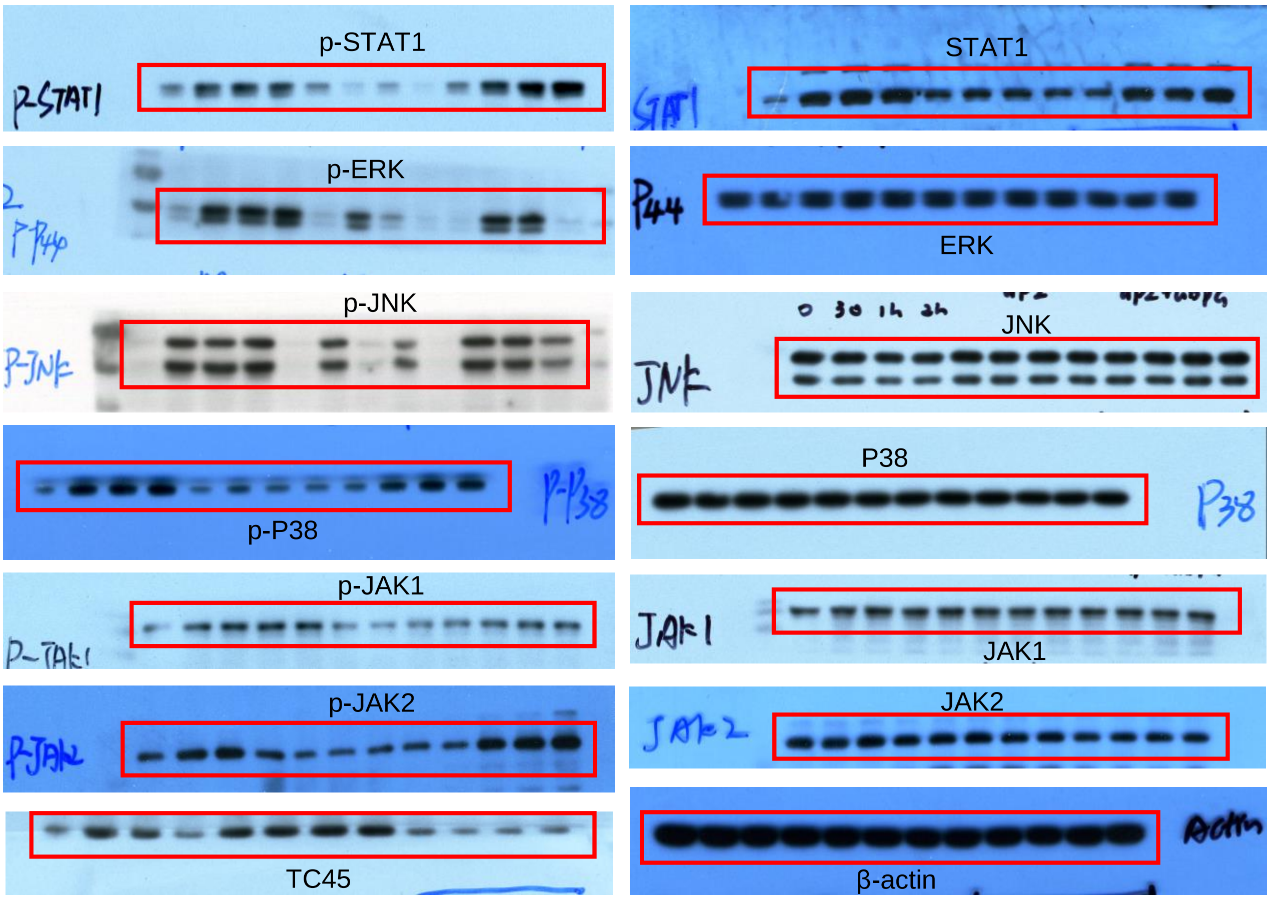

Figure S6b

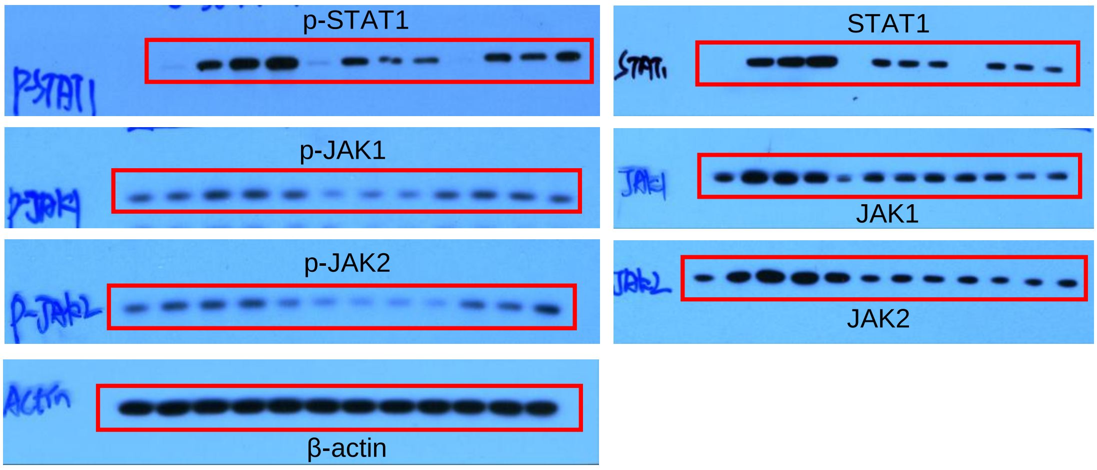

Figure S6c

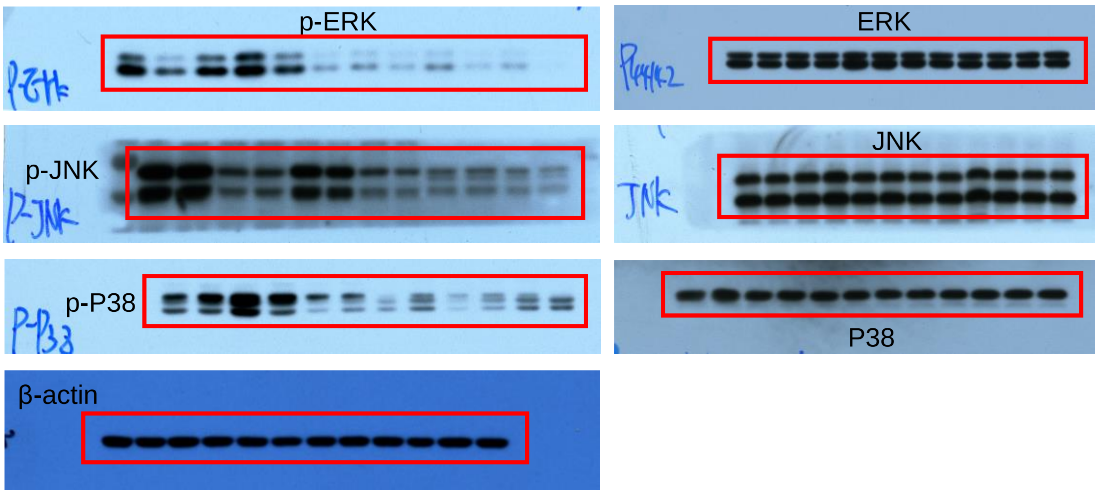

Figure S6d

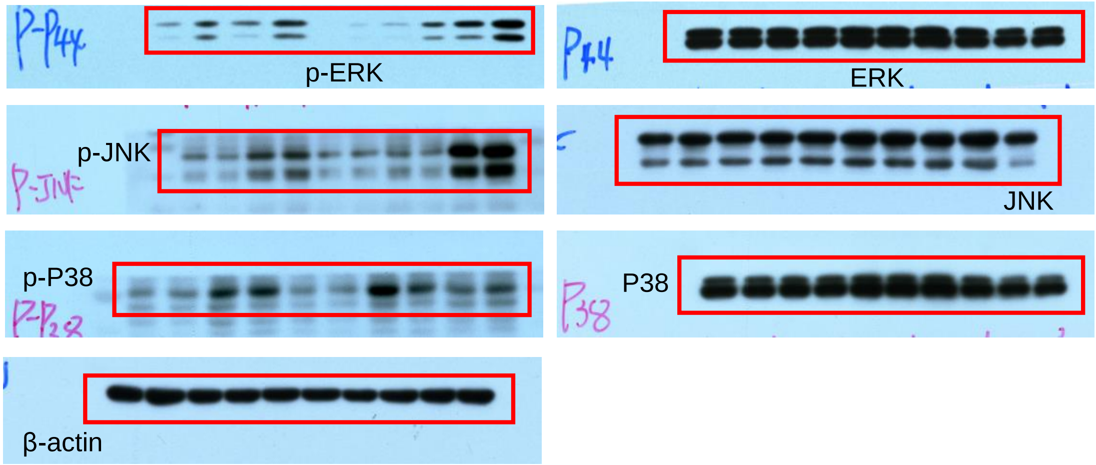

Figure S7h

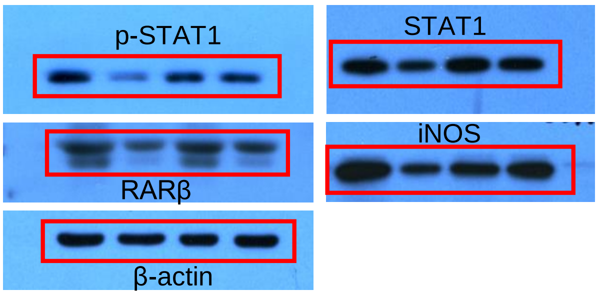

Supplement: Supplementary file 1 — Supplementary Information [file 41467_2020_15636_MOESM1_ESM.pdf]
