# Supplementary material for: Glycogen metabolism regulates macrophage-mediated acute inflammatory responses
Source: Nat Commun. 2020 Apr 14;11:1769. doi: 10.1038/s41467-020-15636-8 (PMC7156451; doi:10.1038/s41467-020-15636-8)
Supplement: Supplementary file 2 — Reporting Summary [file 41467_2020_15636_MOESM2_ESM.pdf]

nature research

Corresponding author(s): Bo Huang

Last updated by author(s): Feb 28, 2020

Reporting Summary

Nature Research wishes to improve the reproducibility of the work that we publish. This form provides structure for consistency and transparency in reporting. For further information on Nature Research policies, see Authors & Referees and the Editorial Policy Checklist .

Statistics

For all statistical analyses, confirm that the following items are present in the figure legend, table legend, main text, or Methods section.

n/a	Confirmed	
	x	The exact sample size (*n*) for each experimental group/condition, given as a discrete number and unit of measurement
	x	A statement on whether measurements were taken from distinct samples or whether the same sample was measured repeatedly
	x	The statistical test(s) used AND whether they are one- or two-sided<br>*Only common tests should be described solely by name; describe more complex techniques in the Methods section.*
x		A description of all covariates tested
x		A description of any assumptions or corrections, such as tests of normality and adjustment for multiple comparisons
	x	A full description of the statistical parameters including central tendency (e.g. means) or other basic estimates (e.g. regression coefficient) AND variation (e.g. standard deviation) or associated estimates of uncertainty (e.g. confidence intervals)
	x	For null hypothesis testing, the test statistic (e.g. *F*, *t*, *r*) with confidence intervals, effect sizes, degrees of freedom and *P* value noted<br>*Give P values as exact values whenever suitable.*
x		For Bayesian analysis, information on the choice of priors and Markov chain Monte Carlo settings
x		For hierarchical and complex designs, identification of the appropriate level for tests and full reporting of outcomes
	x	Estimates of effect sizes (e.g. Cohen's *d*, Pearson's *r*), indicating how they were calculated

Our web collection on statistics for biologists contains articles on many of the points above.

Software and code

Policy information about availability of computer code

Data collection	Confocal images were observed under confocal microscope (Leica SP8) and captured using LAS X Life Science Software, version: 3.0.16120.2 (Buffalo Grove, IL 60089 United States).<br>Real-time PCR was performed on a Bio-Rad CFX Connect and the data was captured using Bio-Rad CFX Manager 2.0 software.<br>Metabolism analysis data were captured using the Xcalibur™ software, version:3.0 (Thermo Fisher).
Data analysis	GraphPad Prism 8 (Generating graphs and performing statistics), Xcalibur Qual browser 3.0 (Analyzing metabolism results).

For manuscripts utilizing custom algorithms or software that are central to the research but not yet described in published literature, software must be made available to editors/reviewers. We strongly encourage code deposition in a community repository (e.g. GitHub). See the Nature Research guidelines for submitting code & software for further information.

Data

Policy information about availability of data

All manuscripts must include a data availability statement. This statement should provide the following information, where applicable:

- Accession codes, unique identifiers, or web links for publicly available datasets
- A list of figures that have associated raw data
- A description of any restrictions on data availability

Source data for Figs. 1b, d, g-j, l-o, 2a, c-e, g-j, 3a-k, m, n, 4a-j, l, n, p, 5a, b, e, g, h, j, l, n, o, q, r, 6l, 7a-d, f-m, o, 8a-d, f, h-n and Supplementary Figs. S1a, b, d-f, S2a, c, e-g, S3a-g, S4b-g, i, j, l, S5b-f, S7a-d, f, g, i, S8a, b and S9 have been provided as Supplemental information. Uncropped western blots for Fig. 1e, k, 2b, f, 3l, 4e-g, k, m, o, 5c, d, k, m, p, 6a, b, d-k, 7n, 8e, g and Supplemental Figs. S1c, g, S2b, d, S4a, f, h, k, S5a, g, S6a-d and S7h have been provided as Supplemental information. All other data supporting the findings of this study are available from the corresponding author on reasonable request.

Field-specific reporting

Please select the one below that is the best fit for your research. If you are not sure, read the appropriate sections before making your selection.

☒ Life sciences ☐ Behavioural & social sciences ☐ Ecological, evolutionary & environmental sciences

For a reference copy of the document with all sections, see nature.com/documents/nr-reporting-summary-flat.pdf

Life sciences study design

All studies must disclose on these points even when the disclosure is negative.

Sample size	At least three biological replicates were achieved for all experiments. Such sample sizes are typical for the in vitro experiments. For in vivo experiments, a sample size of n=4-10 mice was used per experimental group.
Data exclusions	No data were excluded from the analyses.
Replication	Experiments were successfully repeated independently at least three times, as indicated in the figures (n experiments). All attempts for replication were successful.
Randomization	For in vitro experiments, bone marrow-derived macrophages or THP-1 cells were divided equally to each group and then treated with drug agents. For animal studies, mice were randomly distributed into group and then earmarked by an independent researcher.
Blinding	The investigators were not blinded to allocation during in vitro experiments and outcome assessment because treatments and different drugs used made it difficult to blind. Data collection and data analysis were performed by different individuals. The investigators were blinded to allocation during in vivo experiments and outcome assessment (The mice were assigned to treatment groups with randomly assigned mouse number, with the key unknown to operators until experiments were completed).

Reporting for specific materials, systems and methods

We require information from authors about some types of materials, experimental systems and methods used in many studies. Here, indicate whether each material, system or method listed is relevant to your study. If you are not sure if a list item applies to your research, read the appropriate section before selecting a response.

Materials & experimental systems

n/a	Involved in the study	
☐	☒	Antibodies
☐	☒	Eukaryotic cell lines
☒	☐	Palaeontology
☐	☒	Animals and other organisms
☐	☒	Human research participants
☒	☐	Clinical data

Methods

n/a	Involved in the study	
☒	☐	ChIP-seq
☒	☐	Flow cytometry
☒	☐	MRI-based neuroimaging

Antibodies

Antibodies used	The primary antibodies were purchased from Cell Signaling Technology: anti-iNOS (D6B6S#13120, 1:1000), anti-phospho-STAT1-Tyr701 (58D6#9167, 1:1000), anti-STAT1 (9H2#9176, 1:1000), anti-JAK1 (D1T6W#50996, 1:1000), anti-phospho-ERK-T202/Y204 (194G2#4377s, 1:1000), anti-ERK (137F5#4695S, 1:1000), anti-phospho-JNK-T183/Y185 (9251S, 1:1000), anti-JNK (9252S, 1:1000), anti-phospho-P38-Thr180/Tyr182 (3D7#9215, 1:1000), anti-P38 (9212S, 1:1000), anti-Fbp1 (D2T7F, 1:1000), anti-G6pdx (#8866, 1:1000), anti-Gys1 (3893S, 1:1000), anti-TC45 (D7T7D#58935, 1: 1000) and anti-IRF-1 (D5E4#8478, 1:1000). The following primary antibodies were purchased from Abcam: anti-Pgm1 (ab192876, 1:1000), anti-Ugp2 (ab154817, 1:1000), anti-Pygl (ab190243, 1:1000), anti-Pygm (ab88078, 1:1000), anti-P2Y14 (ab136264, 1:1000), anti-G6pase (ab83690, 1:1000) and anti-Histone H3 (ab8284, 1:1000). Anti-phospho-JAK1-Y1022/1023 (YP0154, 1:1000), anti-phospho-JAK2-Tyr570 (YP0306, 1:1000) and anti-JAK2 (YT2428, 1:1000) were purchased from Immunoway. Anti-Pck1 (Z6754-Z-AP, 1:1000) was purchased from Proteintech. Anti-6Pgd (A7710, 1:1000), anti-HK1(A1054, 1:1000), anti-HK2(A0994, 1:1000) and anti-HK3(A8428, 1:1000) were purchased from ABclonal. Anti-ZNF148 (QC7801, 1:1000), anti-RARβ (310315, 1:1000) and anti-β-actin (A1978, 1:10000) were purchased from Sigma. The secondary antibody goat anti-rabbit IgG Dylight®594 (ab96885, 1:400) was purchased from Abcam, HRP-goat anti-rabbit and HRP-goat anti-mouse (1:10000) were purchased from EARTH.
Validation	For western blot and immunofluorescence, all antibodies were used as validated by the manufacturer for their specific assay according to their data sheet. In addition, the staining were consistent with the predicted cellular localization of the protein.<br><br>Abcam: Activity, stability and performance are important checks carried out by our laboratories in the US, Europe and China to ensure we produce high quality products. Our stringent quality control and validation processes use a variety of techniques,

including western blot, ICC/IF, IHC, flow cytometry, ELISA, ChIP, IP and peptide array.
CST: Each Cell Signaling Technology (CST) antibody is validated in-house using rigorous standards including multiple experimental controls and, when available, multiple cell types. An antibody lot is only released when CST scientists are convinced of its specificity and sensitivity in the recommended applications.
Sigma, Immunoway, Proteintech, Abclonal and EARCH: antibodies were validated by the manufacturer and independently for specificity within our lab group by western blot.

Eukaryotic cell lines

Policy information about cell lines

Cell line source(s)	Human monocytic THP-1 cells were purchased from the China Center for Type Culture Collection (Wuhan, China) and cultured in complete RPMI-1640 medium containing 10% fetal bovine serum, 10 mM glucose, 2 mM L-glutamine and 100 U/mL penicillin-streptomycin.
Authentication	The cell lines were not authenticated.
Mycoplasma contamination	Cell lines were tested to be free of mycoplasma contamination.
Commonly misidentified lines (See ICLAC register)	No cell lines listed in the paper are in the database of commonly misidentified cell lines.

Animals and other organisms

Policy information about studies involving animals; ARRIVE guidelines recommended for reporting animal research

Laboratory animals	Female wild-type C57BL/6J mice with 5-7 weeks old were purchased from the Center of Medical Experimental Animals of Hubei Province (Wuhan, China) and P2Y14+/- and P2Y14-/- mice were purchased from the Cyagen Biosciences Inc for studies.
Wild animals	The study did not involve wild animals.
Field-collected samples	No field-collected samples were used in this study.
Ethics oversight	All animal experiments were conducted in accordance with a protocol approved by the Animal Care and Use Committee of Tongji Medical College.

Note that full information on the approval of the study protocol must also be provided in the manuscript.

Human research participants

Policy information about studies involving human research participants

Population characteristics	Peripheral blood was obtained from consenting healthy donors and sepsis patients, both male and female, aged 3-71.
Recruitment	Blood samples from patients with sepsis (n=25) or SIRS (n=28) and healthy controls (n=30) were collected in Union Hospital, Huazhong University of Science & Technology, and Technology. The details of patients were presented in the Supplementary Table 1. The diagnostic standard of sepsis and SIRS is based on the previous publication (JAMA. 2016 Feb 23;315(8):801-10).
Ethics oversight	Ethical permission was granted by the Ethics Committee of the Huazhong University of Science and Technology (HUSTTJMU-2019IEC-S373; Wuhan, China). All patients provided written informed consent to participate in the study.

Note that full information on the approval of the study protocol must also be provided in the manuscript.
